# Vps13F links bacterial recognition and intracellular killing in *Dictyostelium*

**DOI:** 10.1111/cmi.12722

**Published:** 2017-02-21

**Authors:** Jade Leiba, Ayman Sabra, Romain Bodinier, Anna Marchetti, Wanessa C. Lima, Astrid Melotti, Jackie Perrin, Frederic Burdet, Marco Pagni, Thierry Soldati, Emmanuelle Lelong, Pierre Cosson

**Affiliations:** ^1^ Department of Cell Physiology and Metabolism, Faculty of MedicineUniversity of GenevaGenevaSwitzerland; ^2^ Vital‐IT, Swiss Institute of BioinformaticsUniversity of LausanneLausanneSwitzerland; ^3^ Department of BiochemistryUniversity of GenevaGenevaSwitzerland; ^4^ Genomic Research Laboratory, Division of Infectious DiseasesGeneva University HospitalsGenevaSwitzerland

## Abstract

Bacterial sensing, ingestion, and killing by phagocytic cells are essential processes to protect the human body from infectious microorganisms. The cellular mechanisms involved in intracellular killing, their relative importance, and their specificity towards different bacteria are however poorly defined. In this study, we used *Dictyostelium discoideum*, a phagocytic cell model amenable to genetic analysis, to identify new gene products involved in intracellular killing. A random genetic screen led us to identify the role of Vps13F in intracellular killing of Klebsiella pneumoniae. *Vps13F* knock‐out (KO) cells exhibited a delayed intracellular killing of K. pneumoniae, although the general organization of the phagocytic and endocytic pathway appeared largely unaffected. Transcriptomic analysis revealed that *vps13F* KO cells may be functionally similar to previously characterized *fspA* KO cells, shown to be defective in folate sensing. Indeed, *vps13F* KO cells showed a decreased chemokinetic response to various stimulants, suggesting a direct or indirect role of Vps13F in intracellular signaling. Overstimulation with excess folate restored efficient killing in *vps13F* KO cells. Finally, genetic inactivation of Far1, the folate receptor, resulted in inefficient intracellular killing of K. pneumoniae. Together, these observations show that stimulation of *Dictyostelium* by bacterial folate is necessary for rapid intracellular killing of K. pneumoniae.

## 1 INTRODUCTION

Phagocytic cells play a key role in the elimination of invading microorganisms in the human body. These cells ingest many different types of bacteria and eliminate them in phagosomes. In neutrophils and macrophages, phagocytosis is accompanied by a burst in the production of superoxide, and the oxidative burst is thought to play a key role in killing ingested bacteria, because free radicals can react with and damage virtually any biological molecule (Silva, 2010). The evidence implicating free radicals in intracellular killing of bacteria is mostly based on the analysis of patients in which NOX2, which produces superoxide ions, is partially or totally inactivated by mutations. Loss of NOX2 activity results in a disease called chronic granulomatous disease (CGD), characterized by an increased susceptibility to infections with fungi and with a subset of catalase‐positive bacteria (Goldblatt & Thrasher, 2000). In addition, it has been observed that neutrophils from CGD patients are less efficient at killing Staphylococcus aureus in vitro (Ellson et al*.*, 2006). It is not clear why certain bacteria and not others are more prone to mount infections in these patients. Oxidative burst and free radical productions were also reported to play important roles to protect macrophages against infection with *Salmonella* (Rushing & Slauch, 2011). Although these observations have brought to light the role of free radicals in the elimination of ingested bacteria, it is also clear that other killing mechanisms must exist: they presumably account for the fact that CGD patients are not prone to infections with all bacteria.

A number of additional mechanisms have been implicated in intracellular killing, in particular exposure to the acidic pH of phagolysosomes and activity of lytic lysosomal enzymes and of antibacterial molecules such as defensins, cathelicidins and histatins (De Smet & Contreras, 2005; Zanetti, 2005). In neutrophils, the myeloperoxidase‐mediated halogenation as well as the cathepsin G, elastase, and proteinase 3 also contribute to the killing of bacteria (Segal, 2005). Other mechanisms such as the generation of DNA and lytic enzymes that complex by dying neutrophils (NETs: Neutrophil Extracellular Traps) may in addition account for extracellular killing of bacteria (Papayannopoulos & Zychlinsky, 2009). The relative importance of these different killing mechanisms is not fully known, and it is also not clear if different bacteria are killed by different mechanisms. It has for example been shown that elastase knock‐out mice are highly susceptible to infections with Candida albicans, Klebsiella pneumoniae, and Escherichia coli but not with S. aureus *,* whereas mice lacking cathepsin G were highly susceptible to S. aureus (Belaaouaj et al*.*, 1998; Reeves et al*.*, 2002). In these mice, microbial killing was abolished despite a normal oxidative burst, suggesting that free radicals and other antimicrobial mechanisms act synergistically, and that their relative importance in the control of infections depends on the infecting pathogen.

*Dictyostelium discoideum* is a free‐living unicellular organism continuously engaged in bacterial ingestion and killing. Its haploid genome makes it easily amenable to genetic analysis, and it has been used to study many facets of cell biology, in particular cellular motility, phagocytosis, and organization of the endocytic pathway. In addition, *Dictyostelium* provides a good model to study interactions between phagocytic eukaryotic cells and pathogenic or nonpathogenic bacteria (Cosson & Lima, 2014; Cosson & Soldati, 2008). Characterization of mutants with decreased ability to kill ingested bacteria allowed the identification of new gene products involved in intracellular bacterial killing. For example, Kil2, a phagosomal P‐type ATPase presumably transporting Mg^2+^ ions into the phagosome, is essential for intracellular killing of K. pneumoniae bacteria (Lelong et al*.*, 2011). *Kil2* knock‐out (KO) cells still kill efficiently ingested *Pseudomonas aeruginosa* or *Bacillus subtilis*, suggesting that different bacteria are killed by different mechanisms (Lelong et al*.*, 2011).

In this study, we isolated a new *Dictyostelium* mutant defective for intracellular killing of K. pneumoniae. Detailed analysis revealed that *vps13F* KO cells are partially defective in bacterial recognition and as a consequence, fail to efficiently kill ingested K. pneumoniae bacteria. These results provide the first evidence that over a time scale of a few minutes, recognition of ingested bacteria is necessary to ensure efficient intracellular killing.

## 2 RESULTS

### 2.1 Vps13F is involved in the interaction between *Dictyostelium* and Klebsiella pneumoniae

We previously identified Kil2 as a gene product essential for efficient intracellular killing of nonpathogenic, noncapsulated K. pneumoniae (Lelong et al*.*, 2011). In order to identify new gene products involved in intracellular killing of bacteria that could potentially exhibit a functional redundancy with Kil2, we created, in *kil2* KO cells, a library of random mutants by restriction enzyme‐mediated insertion (REMI). We then tested individual clones for their ability to grow on six different nonpathogenic bacteria (*Micrococcus luteus*, B. subtilis, E. coli B/r, K. pneumoniae, K. pneumoniae LM21, and P. aeruginosa) and selected double mutants defective for growth on at least one bacteria. This study is dedicated to the analysis of one mutant strain initially seen to exhibit a defect for growth on K. pneumoniae. The mutagenic vector recovered from this strain, together with the flanking genomic sequences, was found to be inserted in the *vps13F* gene (Figure S1A). In order to ascertain that the growth defect of this original insertional mutant strain was caused by the disruption of the *vps13F* gene, we deleted in the parental strain a large portion of the *vps13F* gene by homologous recombination. Three individual *vps13F* KO clones were selected (Figure S1 B, C, and D), and they all exhibited similar phenotypes, detailed below. Three clones of double *kil2–vps13F* KO were also generated and analyzed in parallel.

**Figure 1:**
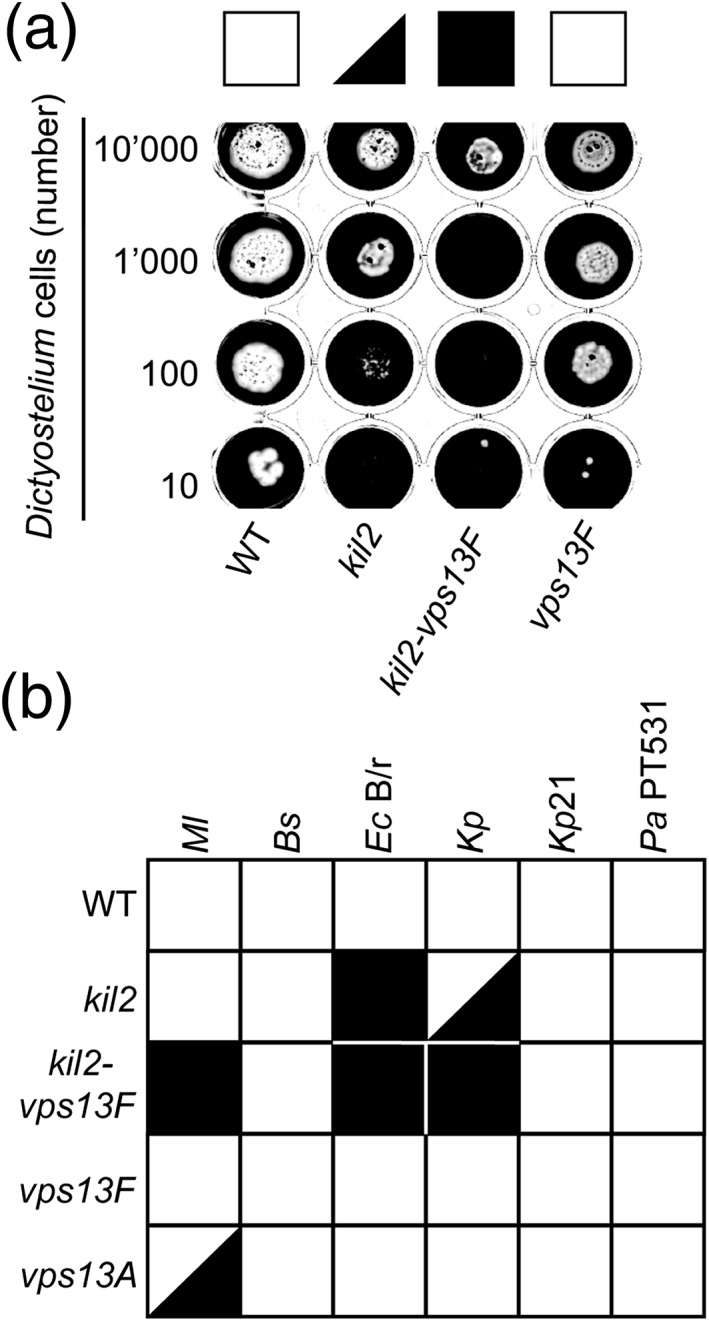
*Vps13F* and *vps13A* KO cells exhibit specific growth defects in the presence of different bacteria. (a) To quantify the ability of *Dictyostelium* mutants to feed upon Klebsiella pneumoniae, *Dictyostelium* cells (10,000, 1,000, 100, or 10 cells) were applied onto a lawn of K. pneumoniae. After five days, wild‐type (WT) *Dictyostelium* cells created phagocytic plaques (white) in the bacterial lawn. *Kil2* KO cells grew slower than WT cells on K. pneumoniae and double *kil2–vps13F* KO cells presented an even more pronounced growth defect. *Vps13F* KO cells grew slightly slower that WT cells, but this growth defect is less pronounced than that of *kil2* KO cells. (b) Growth of Dictyostelium cells on several bacterial species was assessed as shown in (a) in four independent experiments and scored from 4 (efficient growth) to 0 (no growth). A white square indicates an average score of 4–3, a black triangle a score of 3–2, and a black square a score of 2–0. Double *kil2–vps13F* KO cells presented a severe growth defect on Escherichia coli *B/r,* K. pneumoniae and M. luteus. *Vps13A* KO cells grew poorly on M. luteus. (Bs = *Bacillus subtilis*; Ec B/r = Escherichia coli B/r; Kp = K. pneumoniae; Kp21 = Klebsiella pneumoniae LM21; Ml = *Micrococcus luteus*, Pa PT531 = *Pseudomonas aeruginosa* PT531)

We first compared the ability of *kil2‐vps13F* KO cells to grow in the presence of K. pneumoniae with that of its parental single *kil2* KO. For this, a defined number of *Dictyostelium* cells (from 10 to 10,000) was deposited on a lawn of K. pneumoniae bacteria, and *Dictyostelium* growth was observed after 5 days (Figure 1A). Wild‐type (WT) *Dictyostelium* cells grew rapidly in the presence of K. pneumoniae, and *kil2* KO cells grew less efficiently, as previously described (Lelong et al*.*, 2011). When combined with a mutation in *kil2*, disruption of the *vps13F* gene created a strong additional growth defect (Figure 1A). In a WT background, *vps13F* inactivation only slightly delayed growth on K. pneumoniae (Figure 1A). When tested on a wider array of bacteria, the growth defect created by *vps13F* inactivation in the *kil2* KO background was seen when cells were exposed to K. pneumoniae, to a mucoid strain of E. coli B/r, and to M. luteus (Figure 1B). Growth of all these KO strains was identical to that of WT cells in liquid HL5 medium, suggesting that the genetic inactivation of *vps13F* created a specific defect in the interaction of *Dictyostelium* cells with K. pneumoniae and a few other bacteria.

Interestingly, a separate genetic screen performed in a WT background also yielded a mutation in a gene of the Vps13F family (Figure S2): *vps13A* KO cells grew inefficiently in the presence of M. luteus but as efficiently as WT cells in the presence of other bacteria (Figure 1B). This indicates that different members of the Vps13F family have distinct functions during *Dictyostelium* growth in the presence of different bacteria.

Although mutations in vacuolar protein sorting 13 (*Vps13*) genes have already been studied in a number of organisms, the function of Vps13 proteins is still poorly understood. Vps13 was first identified in *Saccharomyces cerevisiae*, which encodes a single member of the family. Its mutation causes a defect in the sorting of lysosomal enzymes, and more specifically, in vesicular transport between the vacuole and the Golgi apparatus (Brickner & Fuller, 1997, Redding, Brickner, Marschall, Nichols, & Fuller, 1996). The human Vps13 family is composed of four members: Vps13A (also called Chorein), Vps13B, Vps13C, and Vps13D. Mutations in Vps13A and Vps13B result in rare neurological diseases, respectively, chorea‐acanthocytosis (Rampoldi et al*.*, 2001; Ueno et al*.*, 2001) and Cohen syndrome (Kolehmainen et al*.*, 2003). In both cases, the large size of these proteins and the lack of well‐characterized domains or motifs in their structure made their functional characterization difficult. To date the molecular function of Vps13 proteins is still poorly understood, as well as their specific involvement in these genetic diseases. The *Dictyostelium* Vps13 family comprises six members, and one of them, Vps13C (also called TipC), was proposed recently to play a role in autophagy (Munoz‐Braceras, Calvo, & Escalante, 2015).

**Figure 2:**
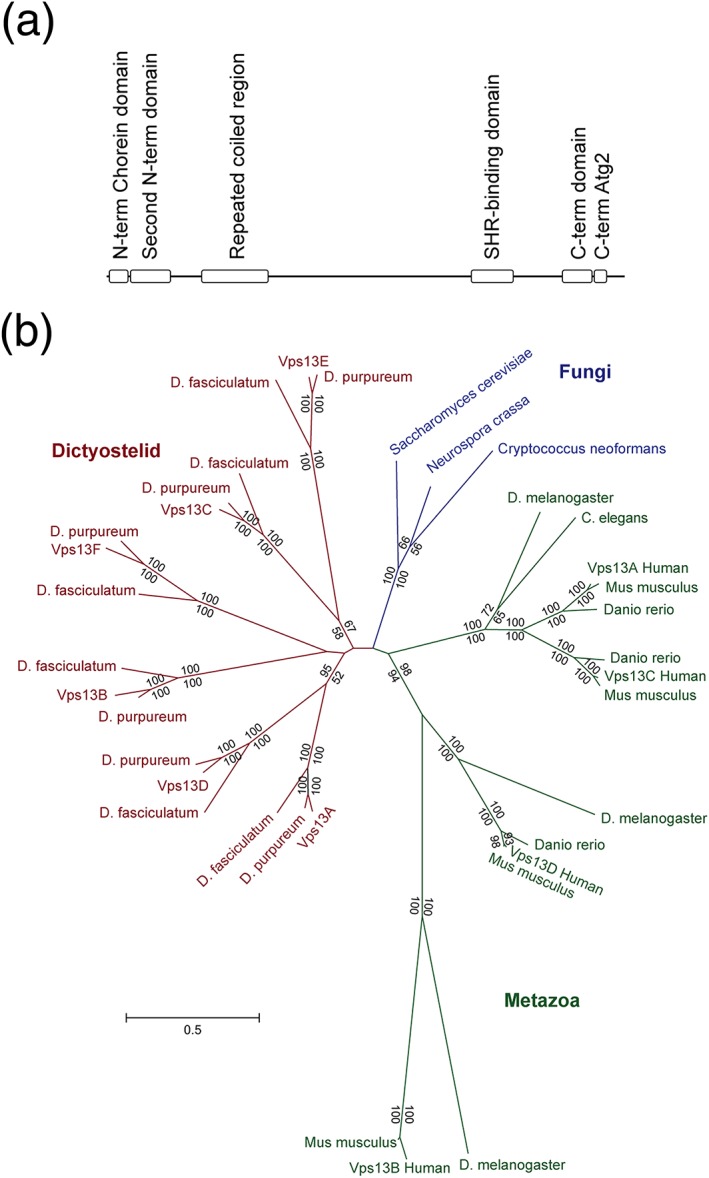
The Vps13F family. (a) Proteins of the vacuolar protein sorting 13 family share a similar domain organization. On the basis of primary sequence analysis, *Dictyostelium* proteins share the same six conserved domains present in human and yeast proteins: two N‐terminal domains (the first one corresponding to the Chorein domain), a repeated coiled region, an SHR‐binding domain, and two C‐terminal domains (the last one corresponding to an autophagy or Atg2‐related domain). (b) Unrooted maximum‐likelihood phylogenetic tree of Vps13F proteins from dictyostelid*,* fungi, and metazoan species. Numbers at the nodes indicate the percentage of bootstrap support (upper values for the maximum‐likelihood tree and lower values for the neighbor‐joining tree; only numbers above 50% are shown)

The six *Dictyostelium* Vps13 proteins show relatively low overall primary sequence similarity with each other (between 25% and 40%), but their domain structure is conserved and essentially identical to that of human and yeast proteins (Figure 2A), with six conserved domains: (a) an N‐terminal domain, also called Chorein domain, (b) a second N‐terminal domain, (c) a repeated coiled region, (d) an an SHORT‐ROOT (SHR)‐binding domain shown in plants to bind the SHR transcription factor (Koizumi & Gallagher, 2013), (e) a C‐terminal domain, and (f) a second C‐terminal autophagy‐related domain (also found in the Atg2 protein). The molecular functions of these domains remain essentially unelucidated in all species. Vps13 proteins have no transmembrane domain or signal peptide sequences, suggesting that they are neither secreted nor present in intracellular organelles.

Phylogenetic reconstructions using the proteins from *Dictyostelium*, human, and yeast suggest that paralogs were generated by duplication independently after divergence of Amoebozoae and of Metazoa (Figure 2B). Each of the six *D. discoideum* paralogs have orthologs in two other *Dictyostelium* species (D. purpureum and D. fasciculatum), indicating that the duplications occurred before the speciation of the Dictyostelid group. *Dictyostelium* Vps13A and D are close, as well as C/E and B/F. The human proteins show a large degree of divergence, as evidenced by the long branch lengths between the four orthologs (Figure 2B).

### 2.2 Defective killing of K. pneumoniae in *vps13F* KO cells

**Figure 3:**
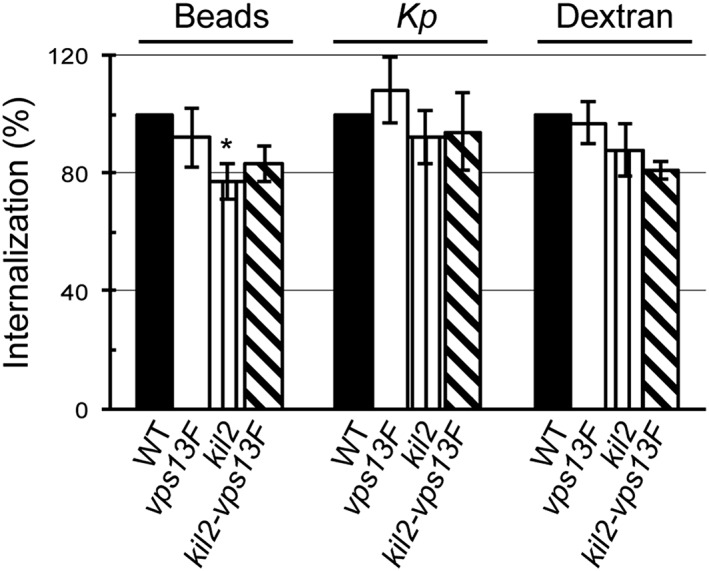
Phagocytosis and macropinocytosis are not defective in *vps13F* KO cells. Wild‐type (WT) or knock‐out (KO) cells were incubated for 20 min with fluorescent latex beads, heat‐inactivated Klebsiella pneumoniae, or dextran. The internalized fluorescence was measured by flow cytometry. Mean fluorescence was plotted for each strain and expressed as a function of internalization in WT cells. Macropinocytosis of dextran and phagocytosis of beads or bacteria were as efficient in *vps13F* KO as in WT cells (mean ± SEM; 5 and 7 independent experiments for WT and KO respectively). *Kil2–vps13F* KO cells exhibited a minor defect in phagocytosis of latex beads compared to WT cells, as also seen in *kil2* KO cells (*p* < .05)

In order to test the putative role of Vps13F in phagocytosis and macropinocytosis, WT and *vps13F* KO cells were incubated in the presence of fluorescent latex beads, of fluorescently‐labeled K. pneumoniae, or of a fluorescent dextran. Phagocytosis of latex beads and of K. pneumoniae was unaffected in *vps13F* KO cells compared to WT cells, as well as fluid‐phase uptake of dextran by macropinocytosis (Figure 3). *Kil2* KO and *kil2–vps13F* KO cells both showed a minor defect in phagocytosis of latex beads compared to WT cells (Figure 3).

**Figure 4:**
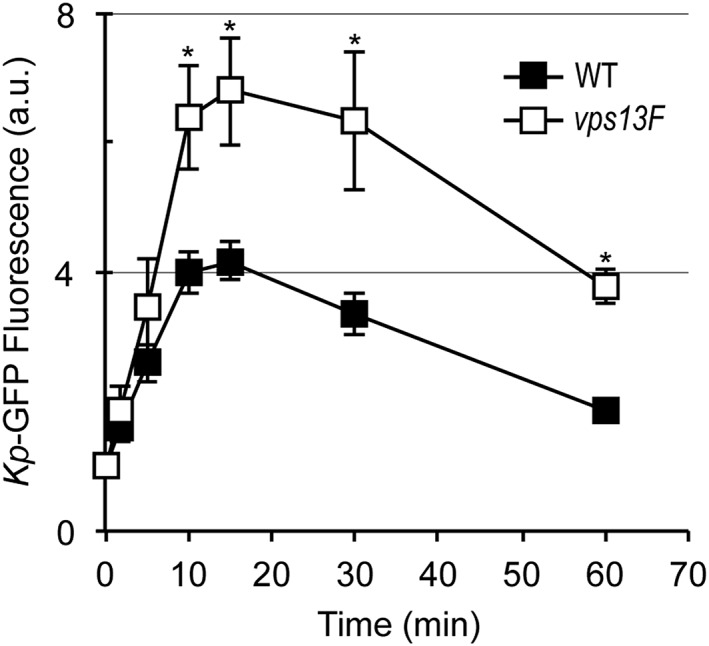
Intracellular accumulation of live *Klebsiella pneumoniae* in *vps13F* KO cells. Wild‐type or *vps13F* KO *Dictyostelium* cells were incubated in the presence of K. pneumoniae expressing GFP (10 bacteria per *Dictyostelium*). At the indicated time, an aliquot was collected, and the fluorescence associated with cells was determined by flow cytometry. The number of intracellular fluorescent bacteria increased gradually and reached a plateau after 15 minutes, when internalization and killing rates equilibrated. Intracellular fluorescence then decreased gradually as extracellular bacteria were depleted. *Vps13F* KO cells accumulated more intracellular fluorescence than WT cells, suggesting that genetic inactivation of *vps13F* causes a defect in intracellular killing of K. pneumoniae. (mean ± SEM; *: *p* < .05; student *t* test; *n* = 7)

We next tested whether ingested bacteria were efficiently killed in *vps13F* KO cells. For this, we followed the fate of internalized K. pneumoniae bacteria expressing Green Fluorescent Protein (GFP) (*Kp*‐GFP). As described previously (Benghezal et al*.*, 2006; Lelong et al*.*, 2011), killing of *Kp*‐GFP bacteria results in extinction of the GFP fluorescence. We first incubated *Dictyostelium* cells with an excess of *Kp*‐GFP bacteria (10 bacteria per *Dictyostelium*) and measured the intracellular accumulation of fluorescent bacteria by flow cytometry. In WT cells, accumulation of intracellular fluorescent bacteria reached a maximum after approximately 15 min, then decreased as extracellular bacteria were gradually ingested and killed (Figure 4). In *vps13F* KO cells, the maximal intracellular fluorescence was also reached after 15 min, but it was significantly higher than in WT cells (Figure 4), suggesting that internalized bacteria remained fluorescent longer in *vps13F* KO cells than in WT cells.

**Figure 5:**
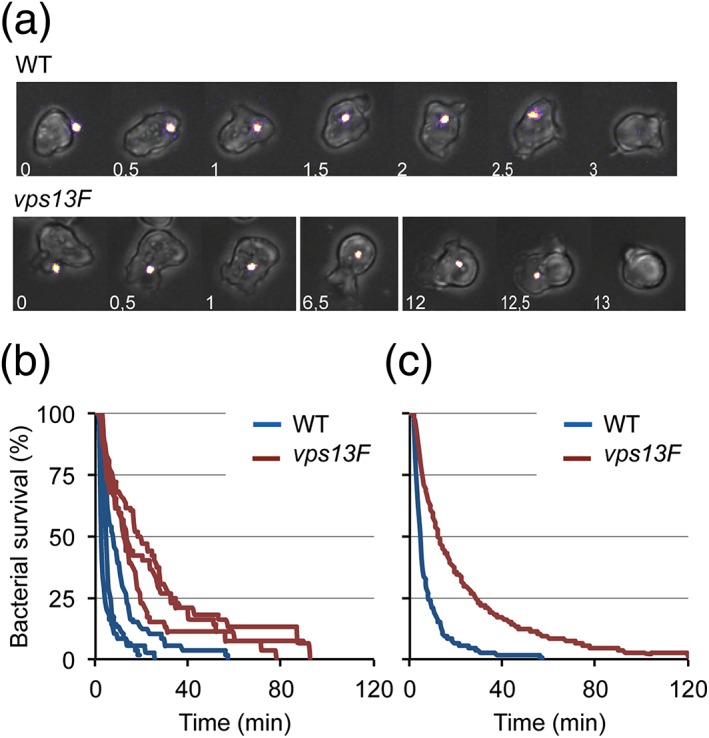
**Impaired intracellular killing of** ***Klebsiella pneumoniae*** **in *vps13F* KO cells.** To visualize ingestion and intracellular killing of individual K. pneumoniae ***,*** *Dictyostelium* cells were incubated with GFP‐expressing K. pneumoniae (*Kp‐*GFP) at a ratio of 1:3 in PB‐Sorbitol for a total duration of 2 h. Cells were imaged every 30 sec by phase contrast and fluorescence microscopy. (a) Representative, successive images showing a WT cell that kills an individual *Kp‐*GFP in 3 min. Below, representative images showing a *vps13F* KO cell killing a *Kp‐*GFP in 13 min. (b) The time between phagocytosis and fluorescence extinction of each phagocytosed bacterium was determined and the probability of bacterial survival is represented as a Kaplan–Meyer estimator. Survival curves of ingested K. pneumoniae collected in three independent experiments in WT cells (blue) and KO cells (red). (c) Survival curves of ingested K. pneumoniae combining results of three independent experiments in WT cells (blue) and seven in *vps13F* KO (red). Intracellular killing is significantly slower in *vps13F* KO cells compared to WT cells (*p* < 10^−4^; log‐rank test; number of ingested bacteria is 228 for WT and 457 for *vps13F* KO cells)

We next visualized phagocytosis and intracellular killing of individual GFP‐expressing K. pneumoniae, as previously described (Delince et al*.*, 2016). For this, we imaged directly *Dictyostelium* cells phagocytosing and killing *Kp*‐GFP and measured the time between ingestion and killing of individual bacteria. Two representative movies are shown (Figure 5A): in these instances, extinction of GFP fluorescence occurred approximately 3 min after phagocytosis in WT cells, while GFP fluorescence persisted for 13 min in *vps13F* KO cells (Figure 5A). The time required for fluorescence extinction was quantified for a large number of bacteria (>100) in at least three independent experiments and plotted as a Kaplan–Meyer survival curve. The curves generated in three independent experiments for WT and *vps13F* KO cells are shown (Figure 5B), as well as the curves combining the results of the three experiments for the WT and seven experiments for *vps13F* KO (Figure 5C). *Vps13F* KO cells killed internalized bacteria significantly slower than WT cells (Figure 5C). The average survival time of internalized bacteria was 7.6 min in WT and 22.1 min in *vps13F* KO cells. Note that the average survival time (7.6 min for WT cells) is significantly higher than the median killing time (5.0 min) due to the fact that a small number of ingested bacteria remain fluorescent for extended periods of time (>30 min), as previously observed (Delince et al*.*, 2016).

**Figure 6:**
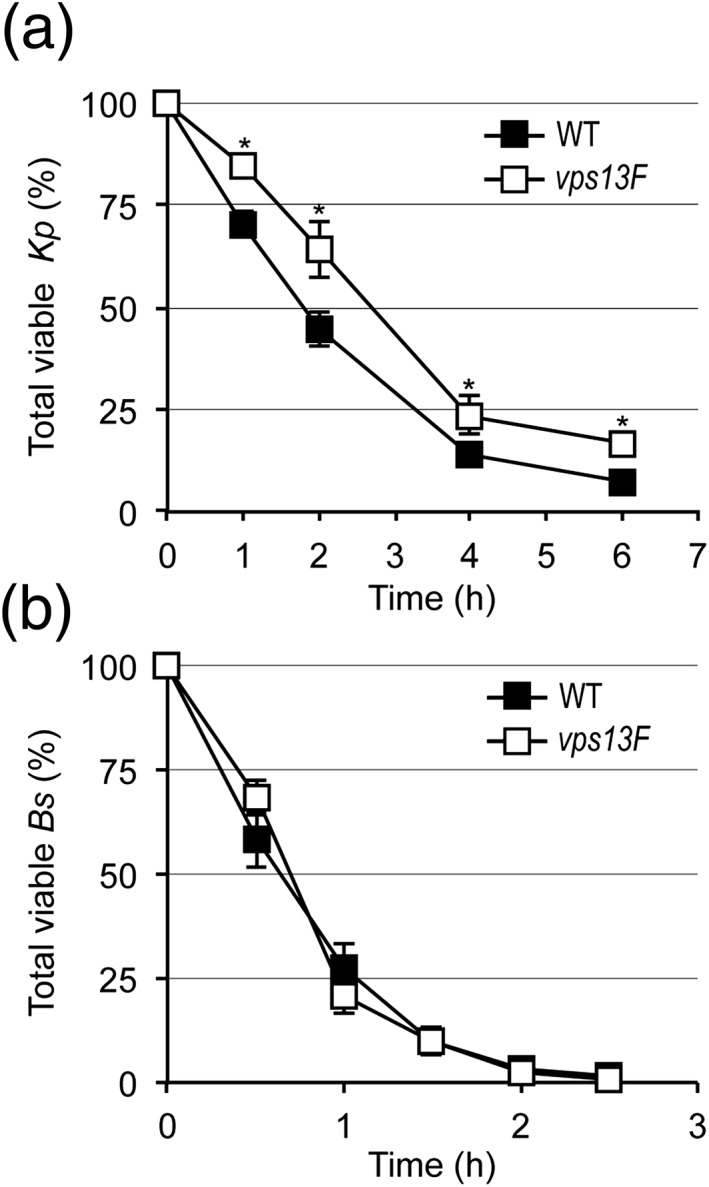
*Vps13F* KO cells are defective for killing *Klebsiella pneumoniae* but not *Bacillus subtilis*. (a) WT or KO *Dictyostelium* were mixed with K. pneumoniae (200 *Dictyostelium* cells per bacteria to ensure optimal phagocytosis). At the indicated times, an aliquot of the mixture was collected, *Dictyostelium* cells were lysed, the bacteria were plated on LB‐agar, and the total (extracellular and intracellular) number of remaining viable bacteria was evaluated by counting colony forming units (CFUs). Results are expressed as a percentage of CFUs at time 0 (mean ± SEM; *: *p* < .01; student *t* test; *n* = 12 for WT or 16 for *vps13F* KO). (b) Intracellular killing of B. subtilis was assessed as described in (a). No significant difference was detected between WT and *vps13F* KO cells (*n* = 4)

Finally, in order to assess directly the viability of bacteria in *Dictyostelium* cells, we incubated WT or *vps13F* KO cells with a small number of K. pneumoniae (200 *Dictyostelium* cells per bacteria) to ensure optimal phagocytosis. At various times, an aliquot of the suspension was collected, the *Dictyostelium* cells were killed, and the surviving (intracellular and extracellular) bacteria plated on agar where they formed colonies after an overnight incubation at 37 °C. WT cells internalized bacteria over a period of 6 h and phagocytosed bacteria are rapidly killed as previously observed (Benghezal et al*.*, 2006; Lelong et al*.*, 2011; Lima, Balestrino, Forestier, & Cosson, 2014; Figure 6A). In *vps13F* KO cells, bacteria survived longer: after 2 h of incubation with *vps13F* KO cells; 64% of bacteria were still alive, against 45% in the presence of WT cells (Figure 6A). Because K. pneumoniae are phagocytosed as efficiently in *vps13F* KO cells and in WT cells (Figure 3), these results confirm the proposal that intracellular killing of K. pneumoniae is delayed in *vps13F* KO cells compared to WT cells. In summary, three different assays indicate that intracellular killing of K. pneumoniae is slower in *vps13F* KO cells than in WT cells. We also tested the survival of B. subtilis upon incubation with *Dictyostelium* cells and observed no difference in survival of ingested B. subtilis in *vps13F* KO cells (Figure 6B).

**Figure 7:**
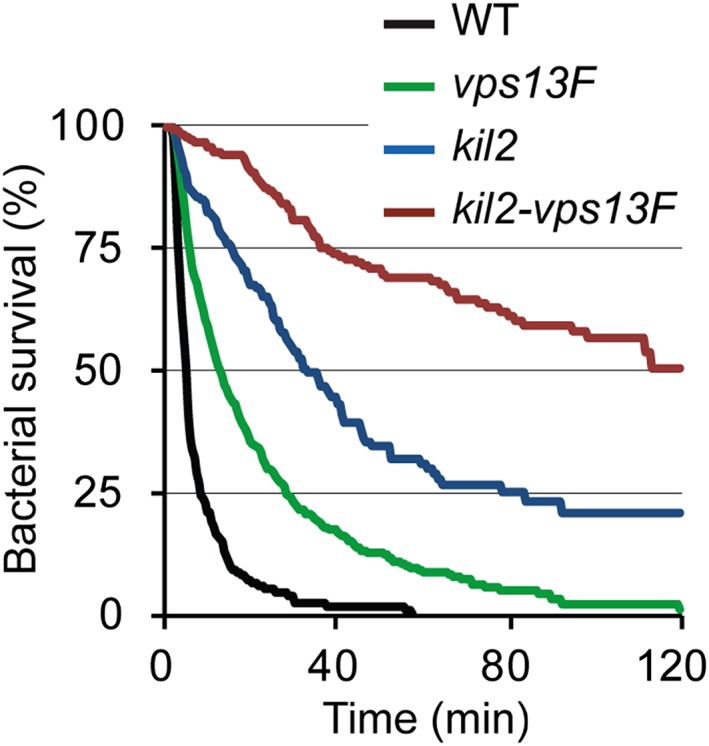
**Deletion of *vps13F* in *kil2* KO cells strongly impacts intracellular killing of** ***Klebsiella pneumoniae*** ***.*** Intracellular killing of individual *Kp*‐GFP by amoeba cells was visualized as described in Figure 5 in WT, *vps13F*, *kil2*, and *kil2–vps13F* KO cells. Intracellular survival of ingested *Kp*‐GFP was significantly longer in *kil2* KO cells than in *vps13F* KO cells, and significantly longer in *kil2–vps13F* KO cells than in *kil2* KO cells (*p* < 10^−4^; log‐rank test; number of ingested bacteria is 198 for *kil2* and 194 for *kil2–vps13F* KO). The sets of data for WT and *vps13F* KO cells are the same as presented in Figure 5C

We then compared the killing defect observed in *vps13F* KO cells with that in *kil2* KO cells: intracellular killing was significantly slower in *kil2* KO cells than in *vps13F* KO cells, and it was even slower in the double *kil2‐vps13F* KO cells (Figure 7; average survival time: 7.6 min in WT cells, 22.1 min in *vps13F* KO*,* 48.5 min in *kil2* KO and 85 min in *kil2‐vps13F* KO cells). The fact that genetic inactivation of *vps13F* conferred an additional killing defect to a *kil2* KO cell suggests that the two proteins function in different pathways in the killing process. Interestingly, *vps13A* KO cells did not exhibit significant defects in ingestion or intracellular killing of K. pneumoniae (Figure S3), confirming the specificity of the *vps13F* KO killing defect. As observed previously with other mutants unable to kill efficiently bacteria (e.g., *kil2* KO cells), both *vps13F* KO and *kil2–vps13F* KO cells grew as efficiently as WT cells in the presence of heat‐killed bacteria (Figure S4).

### 2.3 The organization of the phagocytic and endocytic pathways is not affected in *vps13F* KO cells

**Figure 8:**
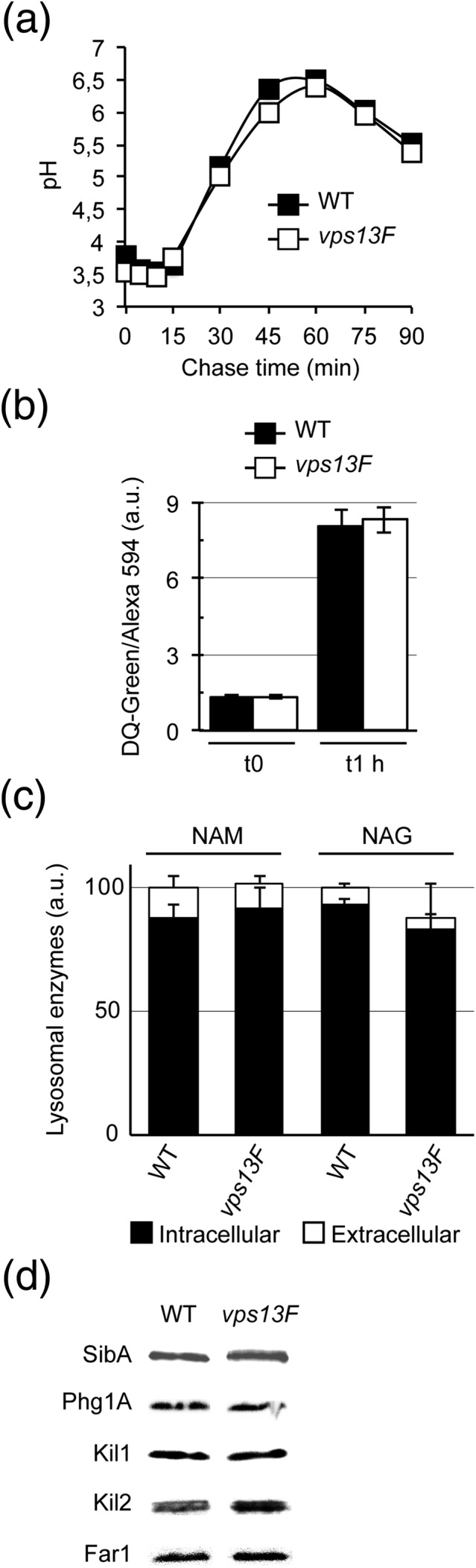
The organization of the endocytic pathway is not altered in *vps13F* KO cells. (a) Kinetics of endosomal acidification and reneutralization in *vps13F* KO and in wild‐type (WT) cells are identical. To assess acidification in endosomal compartments, cells were allowed to engulf for 18 min two fluorescent dextrans, then washed and incubated further for the indicated chase times. Intracellular fluorescence was measured by flow cytometry. The endosomal pH was estimated by the fluorescence ratio of the two internalized probes. This experiment was repeated 3 times with identical results. (b) Phagosomal proteolysis is not defective in *vps13F* KO cells. Cells were incubated with latex beads coupled to BSA labeled with DQ‐green for 15 min, then incubated further for 0 or 1 h. At time 0, internalized beads exhibited low fluorescence. Intracellular proteolysis of BSA released DQ‐green fluorescence and revealed the intra‐phagosomal proteolysis of BSA. Results are expressed as the ratio of DQ‐Green/Alexa‐594 (mean ± SEM; 3 independent experiments). (c) After 3 days of culture in HL5 medium, *Dictyostelium* cells were recovered by centrifugation, and the activity of two lysosomal enzymes (NAG = N‐acetyl β‐glucosaminidase; NAM = α‐mannosidase) was measured in cell pellets and in supernatants using chromogenic substrates. The total activity of lysosomal enzymes was very similar in *vps13F* KO and in WT cells. In both cells, only a small fraction of lysosomal enzymes was secreted (mean ± SEM; 3 independent experiments). (d) Cell lysates of WT and *vps13F* KO cells were migrated on polyacrylamide gels, transferred to nitrocellulose, and analyzed by Western‐blot using antibodies against SibA (209 kDa), Phg1A (55 kDa), Kil1 (56 kDa), Kil2 (131 kDa), and Far1 (70 kDa) proteins (quantification in Figure S7). No significant difference was seen between WT and *vps13F* KO cells

In order to account for the defective killing of K. pneumoniae, we first checked the organization and function of the endocytic and phagocytic pathway in *vps13F* KO cells. Acidification of the endocytic pathway was analyzed by measuring extinction of internalized Oregon green‐labeled dextran as previously described (Marchetti, Lelong, & Cosson, 2009) and was found to be unaffected in *vps13F* KO cells: after an 18‐min pulse, internalized fluid phase was found in the very acidic endosomes, from which it was transferred after approximately 30 min to less acidic postlysosomes (Figure 8A and Figure S5). The morphology of the main endocytic compartments was assessed by immunofluorescence with antibodies against p80, a marker of lysosomes and postlysosomes; p25, a marker of the cell surface and of recycling endosomes; and rhesus, a marker of the contractile vacuole. No gross defects in endosomal morphology and sorting were detected (Figure S6).

Silica beads coated with Bovine Serum Albumin (BSA) coupled to DQ green were used to assess the activity of lysosomal proteases inside phagosomes as previously described (Lelong et al*.*, 2011; Sattler, Monroy, & Soldati, 2013). In *vps13F* KO, like in WT cells, degradation of BSA‐released DQ green and dequenched its fluorescence (Figure 8B). The fact that no difference was seen between WT and *vps13F* KO cells indicates that beads were transferred with similar kinetics to acidic compartments where they were processed by active proteases. Alterations in phagosome maturation and acidification, in protease delivery to phagosomes, or in protease activity would all be expected to delay proteolytic digestion in phagosomes.

We also tested the levels of glycosidases in cells and in the cell supernatant: in WT and in *vps13F* KO cells, intracellular levels of N‐acetyl β‐glucosaminidase and α‐mannosidase were indistinguishable, and only minor amounts of enzymes were detected in the cell supernatant (Figure 8C), indicating that their intracellular sorting was not grossly perturbed.

Finally, we measured by Western blots the intracellular level of several proteins previously shown to participate in phagocytosis and intracellular killing: the cellular levels of SibA (Froquet et al*.*, 2012), Phg1A (Le Coadic et al*.*, 2013), Kil1, and Kil2 were identical in WT and in *vps13F* KO cells, as well as the level of Far1 (Figure 8D and Figure S7).

In summary, all our observations indicate that the organization and function of the endocytic and phagocytic pathways are unaffected by genetic inactivation of *vps13F*, suggesting that the role of Vps13F in intracellular killing of *Klebsiella* is linked to another, more subtle functional alteration.

### 2.4 The killing defect of *vps13F* KO cells is linked to defective bacterial sensing

**Figure 9:**
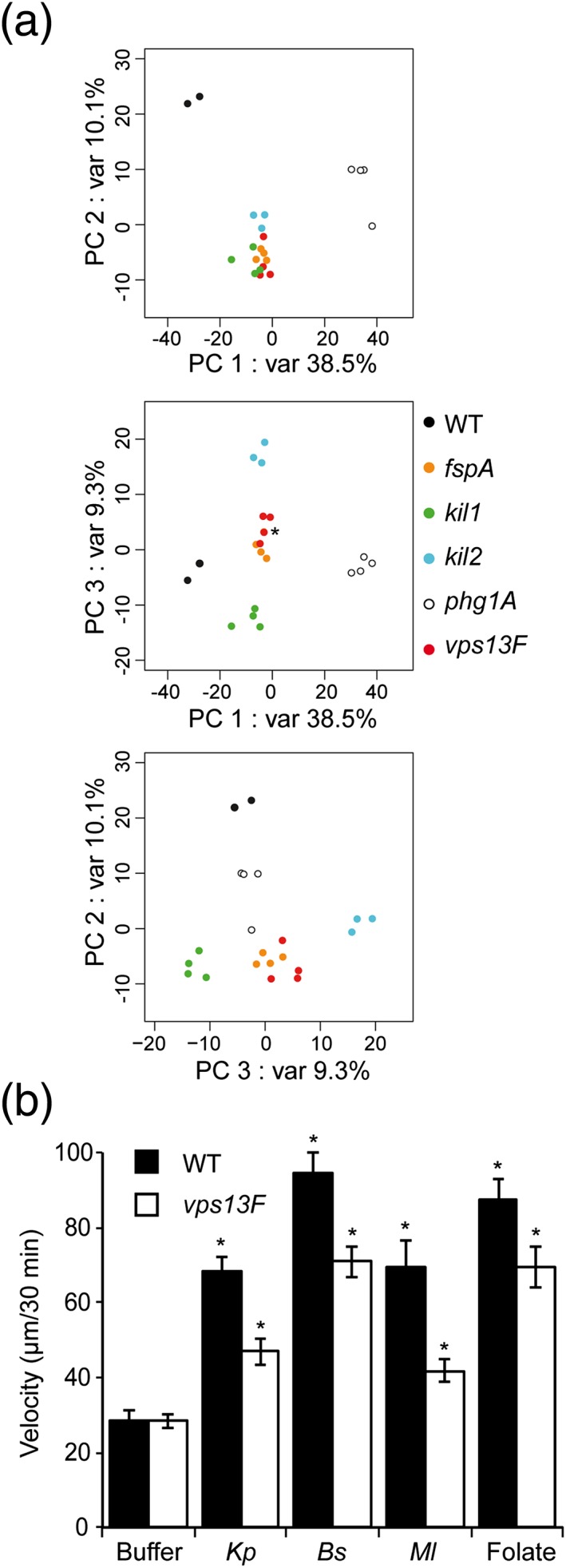
Sensing of bacteria and folate is defective in *vps13F* KO cells. (a) Principal component analysis, done on a subset of 927 genes, showing the three first principal components (explaining 57.9% of the variance). This analysis suggested that *vps13F* KO cells were most closely related to *fspA* KO cells (var = variance; *: the dot corresponding to the fourth *fspA* replicate is hidden behind the *vps13F* dot). (b) Cells were imaged during 30 min in the absence or presence of bacteria (Klebsiella pneumoniae, *Bacillus* subtilis, *Micrococcus* luteus) or folate (1 mM). Individual cell trajectories of 15 cells were tracked to measure cell motility in response to stimulants. Differences between each condition within the same cell type and between *vps13F* KO and WT strains were statistically significant (mean ± SEM; *: *p* < .05; student *t* test; *n* = 14 to 30 independent experiments)

We next analyzed by RNA sequencing the transcription profile of *vps13F* KO cells and compared it to that of WT and of other KO cells defective for growth in the presence of K. pneumoniae: *kil2* (Lelong et al*.*, 2011), *kil1* (Benghezal et al*.*, 2006), *phg1A* (Cornillon et al*.*, 2000), and *fspA* KO cells (Lima et al*.*, 2014). Compared to the experimental variability, the effects of the genetic inactivations were too subtle to be clearly visible from a global expression analysis including all genes. Analysis was thus conducted on a subset of 927 genes, differently regulated in at least one of the pairwise combinations of strains (see experimental procedures). Principal Component Analysis (PCA) revealed that *vps13F* KO cells clustered together with *fspA* KO cells in all three combinations of the first three principal components (Figure 9A), while WT and the other KO cells clustered separately. Although the biological mechanism at play is unclear, this observation suggested that the transcriptional profile of *vps13F* KO cells is more similar to that of *fspA* KO cells than of *kil1*, *kil2*, or *phg1a* KO cells, and led us to analyze if phenotypic traits observed in *fspA* KO cells were also found in *vps13F* KO cells.

Previous results showed that *fspA* KO cells have a defect in sensing folate (Lima et al*.*, 2014), and that folate is the main feature of noncapsulated K. pneumoniae recognized by *Dictyostelium* (Lima et al*.*, 2014). This result led us to test the ability of *vps13F* KO cells to respond to various stimuli.

*Dictyostelium* cells respond to various stimuli by increasing their random velocity. This can be observed after exposure to folate or to different bacteria (K. pneumoniae, B. subtilis, and M. luteus; Lima et al*.*, 2014). In this assay, unstimulated *vps13F* KO cells exhibited a random motility identical to that of WT cells (Figure 9B). Upon stimulation with K. pneumoniae or with M. luteus, a 2.4‐fold increase of motility was observed in WT cells. In these conditions, motility of *vps13F* KO cells also increased significantly (1.5 fold) but to a lesser extent than seen for WT cells (Figure 9B). Addition of B. subtilis or of a high folate concentration (1 mM) increased even more the motility of WT cells (3 fold). *Vps13F* KO cells responded to these more efficient stimulants by increasing 2.4 fold their random motility (Figure 9B). Thus with all stimulants tested, motility of *vps13F* KO cells increased significantly less than observed in WT cells. These experiments suggest that *vps13F* KO cells respond less efficiently to extracellular stimuli, and in particular to Far1‐dependent folate stimulation. Note that strong stimuli (B. subtilis, or a high folate concentration) are sufficient to stimulate *vps13F* KO cells to the same extent as WT cells stimulated with K. pneumoniae or M. luteus.

**Figure 10:**
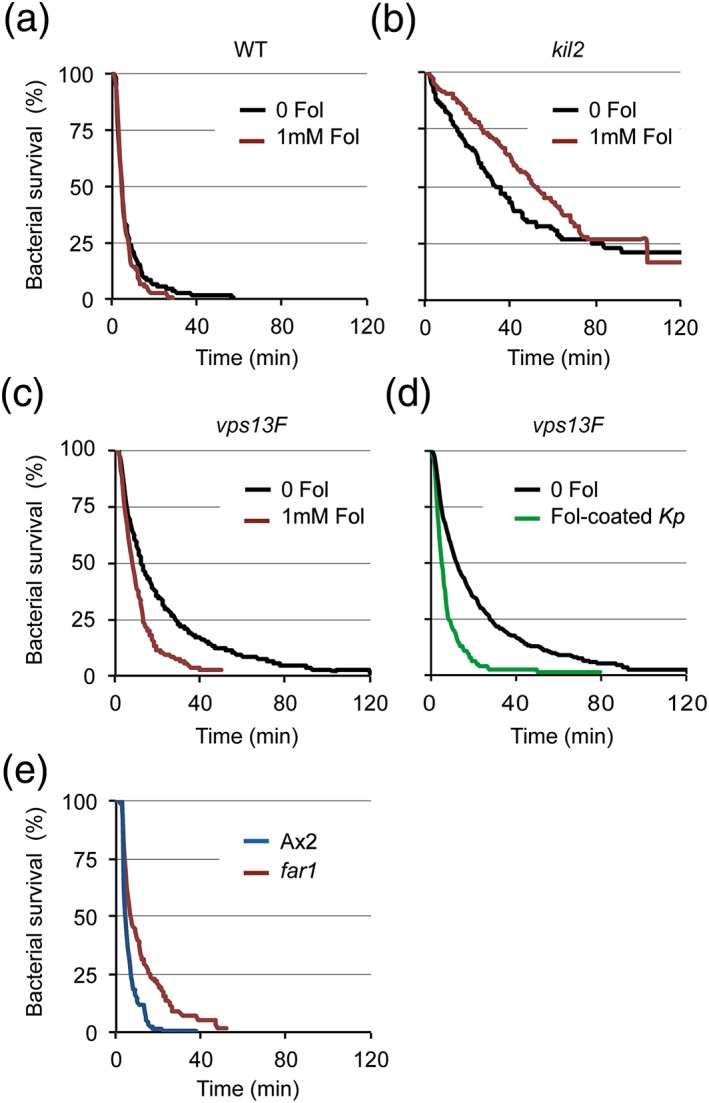
Intracellular killing of *Klebsiella* by *vps13F* KO cells is stimulated by a high concentration of folate. (a‐c). As in Figure 5, intracellular killing of individual *Kp*‐GFP by amoeba cells was visualized in (a) wild‐type (WT) cells, (b) *kil2* KO cells, or (c) *vps13F* KO cells in the presence (red curves) or absence (black curves) of folate (1 mM). In these experiments, folate was directly added in the medium containing cells and bacteria, and imaging was initiated 5 min later. Stimulation with folate significantly accelerated intracellular killing in *vps13F* KO cells but not in WT or *kil2* KO cells (*p* < 10^−4^; log‐rank test; number of ingested bacteria with folate is 350 for *vps13F*, 200 for WT, and 264 for *kil2* KO cells). The sets of data for WT, *vps13F* KO and *kil2* KO cells incubated in the absence of folate are the same as presented in Figure 5c and Figure 7. (d) *Kp*‐GFP bacteria were preincubated for 15 min with 1 mM folate, washed with PB‐Sorbitol, and mixed with *vps13F* KO cells before analysis. In *vps13F* KO cells, intracellular killing of *Kp*‐GFP coated with folate was significantly faster than killing of untreated *Kp‐*GFP (*p* < 10^−4^; log‐rank test; number of ingested bacteria coated with folate is 161). The set of data for killing of untreated *Kp‐*GFP is the same as presented in Figure 5c. (e) Intracellular survival of *Kp*‐GFP in Ax2 cells (blue) and *far1* KO cells (red). Genetic alteration of the Far1 folate receptor significantly impaired intracellular killing of Klebsiella pneumoniae (*p* < 10^−4^; log‐rank test; number of ingested bacteria is 197 for Ax2 and 206 for *far1* KO cells)

We next tested the hypothesis that inefficient killing of *Klebsiella* in *vps13F* KO cells resulted from the inability of *vps13F* KO cells to efficiently respond to the presence of K. pneumoniae. For this, we assessed intracellular survival of K. pneumoniae in cells that were simultaneously exposed to a high concentration of extracellular folate (1 mM). Exposure to folate did not significantly increase killing efficiency in WT cells (Figure 10A), or in *kil2* KO cells (Figure 10B). Strikingly, in *vps13F* KO cells, exposure to folate restored an efficient killing of ingested K. pneumoniae (Figure 10C; average survival time: 22.1 min without folate and 10.7 min in the presence of folate). Note that in this assay, the folate was added to *Dictyostelium* cells at the same time as the bacteria, and a few minutes before imaging. This indicates that the effect of folate on intracellular killing can be observed within minutes of exposure to folate. This result suggests that the slow killing observed in *vps13F* KO cells is due to the fact that these cells are not properly stimulated upon phagocytosis of K. pneumoniae. We next assessed intracellular survival of K. pneumoniae preincubated with folate prior to their ingestion. For this, K. pneumoniae were preincubated with 1 mM folate for 15 min, washed, then incubated with *vps13F* KO cells in the absence of excess folate. In these conditions, efficient killing of ingested K. pneumoniae was restored (Figure 10D; average survival time: 8.23 min for folate‐coated K. pneumoniae). The level of folate secreted by K. pneumoniae is presumably not sufficient to induce efficient killing in *vps13F* KO cells, but a higher level of folate can overcome this sensing defect.

Together, these observations suggest that upon ingestion of K. pneumoniae by *Dictyostelium*, sensing of folate is required to ensure rapid intracellular killing. To test this hypothesis directly, we assessed intracellular killing of K. pneumoniae in cells genetically inactivated for the Far1 folate receptor (Pan, Xu, Chen, & Jin, 2016). Remarkably, *far1* KO cells also showed a significant defect in intracellular killing of K. pneumoniae compared to their parental cell line (Figure 10E; average survival time: 6.1 min in Ax2 and 12.3 min in *far1* KO cells).

## 3 DISCUSSION

In this study, we identified Vps13F as a new gene product involved in intracellular killing of K. pneumoniae bacteria by *Dictyostelium* amoeba. Our key finding, based on the analysis of *vps13F* KO cells, is that efficient intracellular killing of K. pneumoniae requires cells to sense and to respond to the bacteria that they are ingesting. In the case of K. pneumoniae, and as suggested by our previous studies, the main factor allowing *Dictyostelium* cells to sense the presence of K. pneumoniae is the folate secreted by the bacteria.

**Figure 11:**
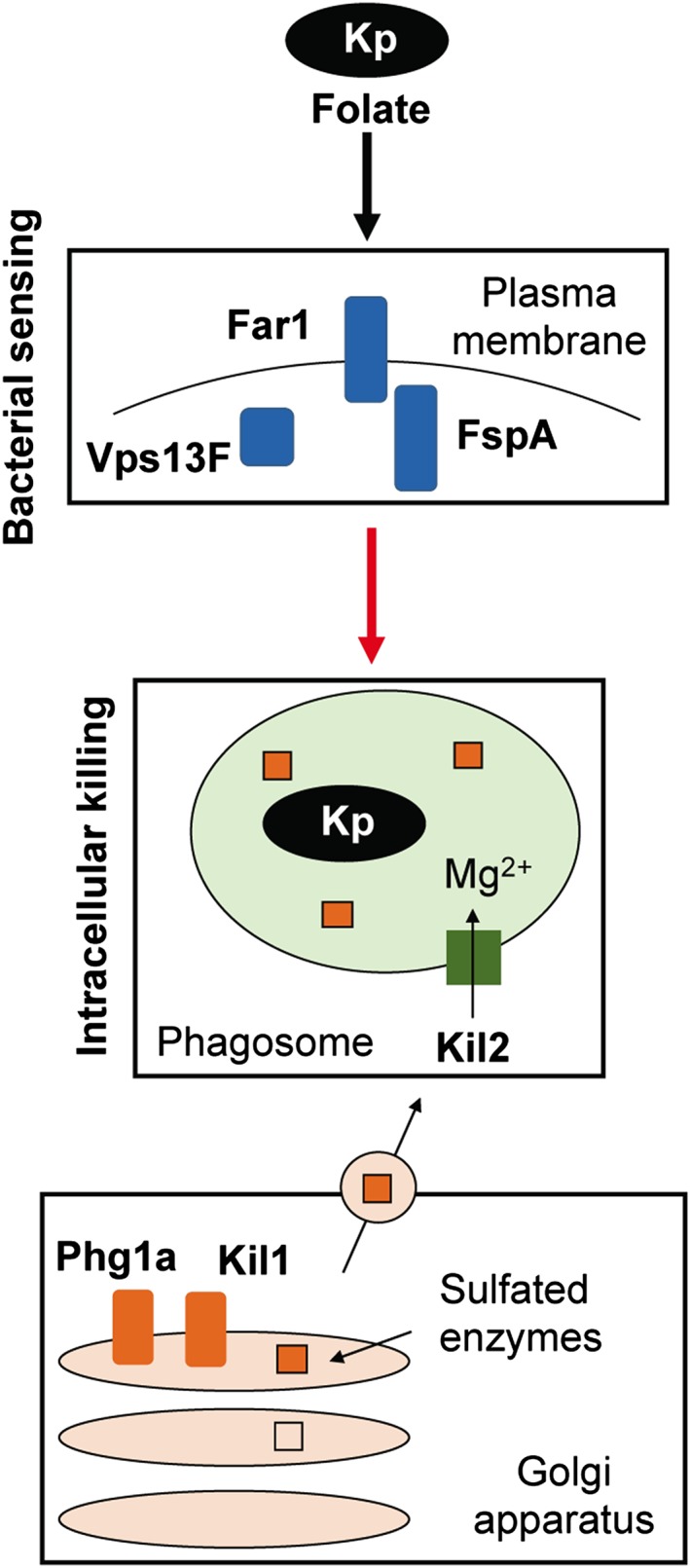
Intracellular killing of *Klebsiella pneumoniae*: a working model. All *Dictyostelium* gene products involved in intracellular killing of K. pneumoniae are depicted in this scheme. In the Golgi apparatus, Phg1 ensures efficient sorting of Kil1, a sulfotransferase sulfating lysosomal enzymes essential for efficient killing. In the phagosome, Mg^2+^ ions transported by Kil2 are necessary for optimal activity of lytic enzymes. The current study indicates that three gene products (Far1, FspA, and Vps13F) are involved in sensing of bacterial folate and are also essential for efficient killing of K. pneumoniae. Identifying the lytic enzymes specifically involved in killing of K. pneumoniae and their regulation is one of our next goals

More specifically, we showed that *vps13F* KO cells respond poorly to extracellular stimulation, suggesting that in these cells inefficient killing of K. pneumoniae is due to a defective sensing of ingested bacteria. Indeed, *vps13F* KO cells overstimulated with a high concentration of folate kill ingested K. pneumoniae as efficiently as WT cells do. Restoration of killing can be achieved by adding folate to the medium during the ingestion of K. pneumoniae, but it is even more efficient to preincubate shortly the K. pneumoniae with folate prior to their encounter with *Dictyostelium*. The critical role of folate sensing in stimulating intracellular killing was further demonstrated by showing that genetic inactivation of the folate receptor also resulted in inefficient intracellular killing of ingested K. pneumoniae. Recent results have shown that *Dictyostelium* senses folate during phagocytosis of bacteria or folate‐coated particles and that this stimulates phagocytosis (Pan et al*.*, 2016). The current study brings these results one step further by showing that after phagocytosis, folate sensing also stimulates the subsequent killing of K. pneumoniae. A working model for intracellular killing of K. pneumoniae bacteria is proposed (Figure 11).

One previous study has shown that *Dictyostelium* cells modify their gene expression patterns following exposure to different bacteria (Nasser et al*.*, 2013). This allows them to adapt to changes in their source of nutrients. However, changes in gene expression occured over a time frame of several hours, while the effect of folate on the kinetics of intracellular killing observed in this study is almost immediate. It is likely that the very rapid sensing of bacteria and slower modifications of gene expression patterns both contribute to ensure optimal adaptation of *Dictyostelium* to changes in its environment and food supply.

Defective growth of *kil2–vps13F* KO cells was apparent in the presence of K. pneumoniae, as well as of a mucoid strain of E. coli (E. coli B/r), and of M. luteus, but not in the presence of other bacterial species like B. subtilis and P. aeruginosa, or even in the presence of another strain of K. pneumoniae (capsulated K. pneumoniae LM21). This phenotype suggests that phenotypic features of a bacterial strain (such as the composition of its cell surface and resistance to various bactericidal mechanisms) are more important than their species to determine their intracellular processing by *Dictyostelium* cells. We only measured the effect of genetic inactivation of *vps13F* on intracellular killing of K. pneumoniae and of B. subtilis, as these are for the moment the only bacteria for which assays measuring intracellular killing have been developed. As observed before with other mutants defective in intracellular killing (*phg1a*, *kil1*, and *kil2* KO cells; Le Coadic et al*.*, 2013; Lelong et al*.*, 2011), alteration of the *vps13F* gene affects intracellular killing of K. pneumoniae, but not of B. subtilis, suggesting that the molecular mechanisms engaged in intracellular killing of different bacteria are largely distinct. Together, these new results reinforce the notion that molecular mechanisms responsible for intracellular killing of bacteria exhibit a high degree of specificity.

Our results do not identify the exact molecular role of Vps13F in response to extracellular stimulants. Because Vps13 has been proposed to play a role in intracellular sorting in S. cerevisiae, one possibility would be that genetic inactivation of *vps13F* in *Dictyostelium* perturbs intracellular transport of one or several molecules critical for sensing. Existing knowledge on the cellular function of Vps13F proteins is, however, very succinct. It is equally possible that the main role of Vps13 is to participate directly in intracellular activation, and that its function in intracellular sorting in S. cerevisiae was an indirect effect of an alteration of intracellular signaling. Our study indicates that a certain degree of specificity exists between different members of the family, because genetic inactivation of *vps13A* and *vps13F* in *Dictyostelium* resulted in radically different phenotypes. More detailed studies will be necessary to determine the exact mode of action of Vps13F proteins.

Concerning the strategy followed in this study, the *vps13F* insertional mutant was identified by screening a library of random mutants generated in a *kil2* KO background. The underlying assumption was that there is a certain degree of redundancy in mechanisms ensuring the intracellular killing of bacteria, and in this case more specifically of K. pneumoniae. Consequently, the role of certain gene products in intracellular killing may become more apparent when other killing mechanisms are inactivated. This hypothesis is confirmed by our results: the *vps13F* insertional mutant would not have been selected if it had been created in a WT background: it exhibits a significant but limited killing defect, which is not sufficient to cause a major growth defect in the presence of K. pneumoniae bacteria. On the contrary, genetic inactivation of *vps13F* in a *kil2* KO background causes a strong additional growth defect in the presence of K. pneumoniae *.* In further studies, analysis of double or triple KO cells may be necessary to determine the role and the relative importance of various gene products in intracellular killing of different types of bacteria.

## 4 EXPERIMENTAL PROCEDURES

### 4.1 Cell culture and strains

*Dictyostelium* cells were grown in HL5 medium at 21 °C (Cornillon, Olie, & Golstein, 1998) and subcultured twice a week to maintain a density below 10^6^ cells/ml.

Unless specified, *Dictyostelium* cells used in this study were all derived from the DH1–10 subclone (Cornillon et al*.*, 2000) of the *D. discoideum* strain DH1 (Caterina, Milne, & Devreotes, 1994), referred to in this study as wild‐type (WT). The *phg1A* (Cornillon et al*.*, 2000), *kil2* (Lelong et al*.*, 2011) and *fspA* (Lima et al*.*, 2014) KO strains were described previously. In this study, we created a new *kil2* KO strain by deleting a sequence of the *kil2* gene in DH1–10 and replacing it with a Blasticidin S Resistance (BSR) cassette (Figure S8). The BSR cassette was then excised by extrachromosomal expression of Cre (Linkner, Nordholz, Junemann, Winterhoff, & Faix, 2012). This new *kil2* KO strain behaved in the same manner as the previously published *kil2* KO strain (Lelong et al*.*, 2011), and it was used as a starting point for mutagenesis (see below). Ax2 and *far1* KO strains were a kind gift of Dr. Miao Pan and Pr. Jin Tian (National Institute of Health, MD, USA; Pan et al*.*, 2016).

Bacterial strains were grown overnight in Lysogeny broth (LB) medium at 37 °C. Bacteria used were uncapsulated K. pneumoniae laboratory strain (Benghezal et al*.*, 2006)*,* capsulated K. pneumoniae LM21 (Balestrino, Ghigo, Charbonnel, Haagensen, & Forestier, 2008), E. coli B/r (Gerisch, 1959) and *P. aeruginosa* PT531 (Cosson et al*.*, 2002), *B. subtilis* 36.1 (Ratner & Newell, 1978), and *M. luteus* (Wilczynska & Fisher, 1994).

### 4.2 Screening for growth‐deficient *Dictyostelium* mutants

To isolate *Dictyostelium* mutants that are specifically unable to grow in the presence of bacteria, *kil2* KO cells were mutagenized by restriction‐enzyme–mediated insertion of the pSC plasmid and screened as previously described (Cornillon et al*.*, 2000; Lelong et al*.*, 2011; Figure S1). Briefly, individual mutant cells were cloned in 96 well plates using a cell sorter. Overall, 10,000 individual clones were tested for their ability to grow efficiently on several bacteria: M. luteus, B. subtilis, E. coli B/r, K. pneumoniae, K. pneumoniae LM21, and P. aeruginosa PT531. Mutants that grew poorly on at least one of the tested bacteria were selected and expanded, and their genomic DNA was extracted. To identify the site of insertion of the pSC plasmid in each mutant, genomic DNA was digested with ClaI, self‐ligated, transformed in E. coli SURE, and sequenced. Mutants in which the plasmid was inserted in a coding region were selected for further analysis. The *vps13F* mutant was isolated in a *kil2* KO background, while the *vps13A* mutant was identified in a WT background (Figures S1 and S2).

A KO plasmid was constructed to replace a sequence in the *vps13F* gene with a BSR cassette, in both WT and *kil2* KO strains (Figure S1), to generate simple *vps13F* KO and double *kil2–vps13F* KO cells. Individual clones were identified by polymerase chain reaction (PCR) (Figure S1). Three independent clones of each KO cells were obtained and yielded identical results in this study.

The plasmid recovered from the *Dictyostelium* genome after plasmid rescue and containing the genomic region flanking the insertion site in *vps13A* was used to create new *vps13A* KO (Figure S2). Three independent clones were obtained, but only one was used in this study.

### 4.3 Growth of *Dictyostelium* in the presence of bacteria

*Dictyostelium* cells were grown in the presence of bacteria as described previously (Froquet, Lelong, Marchetti, & Cosson, 2009). Briefly, 50 μl of an overnight bacterial culture were plated on 2 ml of SM‐agar in each well of a 24‐well plate. Alternatively, to test the growth of *Dictyostelium* in the presence of dead bacteria, an overnight culture of K. pneumoniae (7 ml) was boiled for 4 h at 95 °C, pelleted, resuspended in 200 μl and applied in each well. Then, 10, 100, 1,000 or 10,000 *Dictyostelium* cells were added on top of the bacterial lawn. Growth of *Dictyostelium* generated phagocytic plaques after 4–7 days of incubation at 21 °C. Quantification of the extent of the growth defect was done by scoring the growth of *Dictyostelium* strains on each bacteria in at least four independent experiments. For each experiment, growth was scored from 4 (efficient growth) to 0 (no growth). For each bacteria tested, the average score of *Dictyostelium* growth was calculated.

### 4.4 Phagocytosis and macropinocytosis

To measure efficiency of phagocytosis, 3 × 10^5^ *Dictyostelium* cells were washed once, resuspended in 1 ml of Phosphate Buffer (PB: 2 mM Na_2_HPO_4_, 14.7 mM KH_2_PO_4_, pH 6.5) supplemented with 100 mM sorbitol (PB‐Sorbitol) and incubated for 20 min with 1 μl FITC latex beads (Fluoresbrite plain YG 1 micron, Polysciences), or with 5 × 10^7^ glutaraldehyde‐fixed K. pneumoniae labeled with rhodamine at a multiplicity of infection of 1:200. To assess macropinocytosis, cells were incubated in PB‐Sorbitol containing 10 μg/ml Alexa‐647 Dextran (Life Technologies) for 20 min. Then, cells were washed in ice cold HL5 supplemented with 0.1% NaN_3_ and internalized fluorescence was measured by flow cytometry. Mean fluorescence was plotted for each strain.

### 4.5 Intracellular killing of bacteria

Three methods were used to measure intracellular killing of bacteria. First, as previously described (Benghezal et al*.*, 2006), cells were mixed with a small number of bacteria (200 *Dictyostelium* cells for 1 K. pneumoniae) and incubated at 21 °C in PB‐Sorbitol. Aliquots were taken at different time points, cells were lysed, and bacteria were plated on LB‐agar. The number of colony‐forming units decreased as bacteria were ingested and killed.

A second method (Benghezal et al*.*, 2006) was to incubate *Dictyostelium* cells with a larger number of GFP‐expressing K. pneumoniae (*Kp*‐GFP, 10 bacteria per *Dictyostelium* cells) and to measure by flow cytometry the accumulation of GFP fluorescence in cells. For this, bacteria were grown overnight in LB supplemented with 100 μg/ml of ampicillin, and washed once with PB‐Sorbitol. *Dictyostelium* cells (10^5^) were washed with PB‐Sorbitol, mixed with 10^6^ bacteria in 1 ml PB‐Sorbitol and incubated at 21 °C. At the indicated times (0–60 min), a 100 μl aliquot was collected. Then, cells were washed in ice cold HL5 supplemented with 0.1% NaN_3_ and internalized GFP fluorescence was measured by flow cytometry.

To measure phagocytosis and intracellular killing of individual bacteria (Delince et al*.*, 2016) *Kp*‐GFP bacteria were mixed with *Dictyostelium* cells at a ratio of 3:1 in PB‐Sorbitol, deposited on a glass slide (Fluorodish, World Precision Instruments, Inc.) for 10 min, then imaged every 30 sec for 2 h with a videotime lapse (Zeiss Axiovert 200 M). At each time, one picture (phase contrast and GFP fluorescence) was taken in four successive focal planes (step size 3 μm) to image the whole cell volume. The Metamorph software was used to extract images, and ImageJ to compile and analyze movies. Survival analysis of phagocytosed fluorescent bacteria was computed using the Kaplan–Meier estimator. Statistical comparisons between Kaplan–Meier curves were done using the log‐rank test. We rejected the null hypothesis if the *p* value was below 10^−3^. Statistical analysis was done using XLSTAT (Version 2016.03.31333). For each condition, the number of ingested bacteria is indicated and at least three independent experiments were performed.

### 4.6 Chemokinetic response to folate and bacteria

For chemokinetic measurements, 2 × 10^4^ *Dictyostelium* cells were allowed to attach to the polystyrene bottom of one well of a 96‐well microplate (Cell culture microplate PS F‐bottom, μclear, Greiner bio‐one) for 20 min in 100 μl of PB‐Sorbitol with or without supplementation of 1 mM folate, or of bacteria (1:1000 *v*/v., from an overnight culture washed twice in PB‐Sorbitol). Cells were then imaged every 15 sec during 30 min using the widefield plate reader ImageXpress XL with a 10X S Fluor objective. The images were acquired with a CoolSnap HQ camera (Photometrics) and movies assembled with Metamorph. Track point tool of Metamorph was used to track individual trajectories and total distance of 15 cells for each experiment and to calculate velocity.

### 4.7 Organization of endosomal and lysosomal pathways

Kinetics of endosomal acidification were assessed by flow cytometry as previously described (Marchetti et al*.*, 2009). Endosomal pH was determined by fluorescence levels of two internalized dextrans, one coupled to pH‐sensitive fluorophore and the other coupled to pH‐insensitive fluorophore. The activity of lysosomal glycosidases in cells and in supernatant was measured as previously described (Le Coadic et al*.*, 2013) using a colorimetric assay.

The activity of phagosomal proteases was measured as previously described (Lelong et al*.*, 2011; Sattler et al*.*, 2013), using silica beads coupled to Alexa‐594 red fluorescent succinimidyl ester (Molecular Probes) and to BSA labeled with DQ‐green (490 nm, Molecular Probes) at a self‐quenching concentration. Cells were allowed to engulf beads in phosphate buffer for 15 min, then aliquots collected after 0 or 1 h. Upon proteolysis, green fluorescence was released and measured by flow cytometry.

### 4.8 Immunolabeling

To perform immunofluorescence analysis, 10^6^ cells were let to adhere to a glass coverslip for 30 min in HL5 medium. Then, *Dictyostelium* cells were fixed with 4% paraformaldehyde for 30 min, washed, permeabilized with methanol at −20°C for 2 min, and labeled with the indicated primary antibody in phosphate buffer with 0.2% bovine serum albumin for 1 h. Permeabilized cells were labeled with markers of endosomal compartments (p80, p25) and of the contractile vacuole (Rhesus). Cells were stained with the corresponding Alexa‐488 fluorescent secondary antibodies for 1 h and observed by LSM700 confocal microscopy (Carl Zeiss).

To determine the levels of cellular proteins, 10^6^ cells were resuspended in 10 μl of 0.103 g/ml sucrose, 5 × 10–2 M Tris, pH 6.8, 5 × 10–3 M EDTA, 0.5 mg/ml bromophenol blue, 2% SDS, and proteins were separated by electrophoresis on an SDS‐polyacrylamide gel. Proteins were then transferred to a nitrocellulose membrane for immunodetection using anti‐Phg1A (Blanc, Zufferey, & Cosson, 2014), anti‐SibA (Cornillon et al*.*, 2006), anti‐Kil1 (Benghezal et al*.*, 2006), and anti‐Kil2 (Lelong et al*.*, 2011) primary antibodies. Horseradish‐peroxidase‐coupled antimouse (for anti‐SibA and anti‐Phg1A) and antirabbit (for anti‐Kil1 and anti‐Kil2) antibodies were used as secondary antibodies. A recombinant anti‐Far1 antibody was generated by the Geneva Antibody Facility (http://www.unige.ch/antibodies; reference MRB 168).

### 4.9 Sequence and Phylogenetic analysis

Protein sequences of Vps13F homologs from a diverse group of organisms were aligned using the K‐align algorithm (Lassmann, Frings, & Sonnhammer, 2009). The alignment was then manually refined in order to remove regions that were hyper variable or with gaps. Phylogenetic trees were generated using MEGA 6.0 (Tamura, Stecher, Peterson, Filipski, & Kumar, 2013). Genetic distances were computed using the Jones‐Taylor‐Thornton algorithm, and Neighbor‐Joining (NJ) was used to generate distance‐based phylogenetic trees. Maximum‐likelihood (ML) phylogenetic estimates were obtained with the Le_Gascuel_2008 model. Sequence evolution model was selected using the “find best model option” in MEGA 6.0. Bootstrap assessment of tree topology with 100 replicates was performed to find the support for the inferred clades. Similar topologies were found for the two phylogenetic methods employed; the star‐shaped, unrooted tree displayed in Figure 2B corresponds to the maximum‐likelihood topology (with bootstrap values for both ML and NJ trees shown). The organisms and the accession codes of the proteins investigated in the phylogenetic analysis are shown in Table S2.

### 4.10 RNA sequencing and analysis

RNA was isolated from at least 5 × 10^6^ *Dictyostelium* cells using the Direct‐zol RNA MiniPrep kit (Zymo Research, # R2052). The quality of RNA was confirmed with a Bioanalyzer (Agilent, RNA 6000 Nano Kit # G2938–90037). Libraries were constructed from 100 ng of RNA using the Ovation Universal RNA‐Seq System kit (Nugen, # 0343). The quality of the libraries was verified by TapeStation (Agilent, High Sensitivity D1000 ScreenTape, # 5067–5584). Samples were pooled and run in single read 50 flow cell (Illumina, # 15022187) and run on a Hiseq 2500 (Illumina).

From six different *Dictyostelium* strains, 21 libraries were analyzed: WT (2 replicates), *fspA* (4 replicates), *kil1* (4 replicates), *kil2* (3 replicates), *phg1A* (4 replicates), and *vps13F* (4 replicates) KO cells. 50 nt singe‐end reads were mapped to the *Dictyostelium discoideum* genome (2009, downloaded from dictybase) using tophat (version 2.0.13) and bowtie2 (version 2.2.4) softwares. As the RNASeq data is stranded, parameter library‐type was set to fr‐secondstrand. Multihits were not allowed, by using option ‐‐max‐multihits 1. The other parameters were default. The read counts per gene were generated using HTSeq software (version 0.6.1) and the GFF annotation downloaded from dictybase (February 2015). Options for htseq‐count were ‐t exon ‐‐stranded = yes ‐m union. The counts were then imported in R (version 3.2.2). The genes were filtered for minimal expression, by removing genes with an average through all samples lower than 5 reads. Normalization factors to scale the libraries sizes were calculated using edgeR. The read counts were then log transformed and variance stabilized using voom. The log‐transformed counts were then batch corrected for date effect using the R package sva and the ComBat function. The experimental design (mutation) was provided to the ComBat algorithm.

A differential expression analysis was then performed on these batch‐corrected data using the R package limma. All the comparisons 2 by 2 were performed between the 6 conditions, so in total 15 comparisons. The genes having an adjusted p‐value lower than 0.05 and an absolute log fold change above 1.5 were considered differentially expressed. The union of these genes was then taken for the following of the analysis. The principal component analysis were generated using the R function prcomp, with centering and scaling the data. The 3 first principal components were considered and plotted versus each other.

## Supporting information


**Figure S1**. **Isolation and generation of *vps13F* KO cells.** A. Schematic representation of the *vps13F* insertional mutant obtained by REMI mutagenesis, with the mutagenic plasmid pSC inserted 7′144 nucleotides (nt) after the start codon. B. The site of insertion was identified by digestion of genomic DNA with ClaI, which allowed the recovery of the mutagenic plasmid with the genomic flanking regions of *vps13F*. C. Schematic representation of the *vps13F* gene in WT or KO cells. To create a new *vps13F* KO, we deleted 909 nt of the genomic sequence, 1′752 nt downstream of the *vps13F* start codon and replaced this portion with a blasticidin resistance cassette by homologous recombination. Arrows indicate the positions of the oligonucleotides used to identify KO cells. D‐E. Identification of *vps13F* KO cells was done by PCR using distinct pairs of oligonucleotides to verify both loss and gain of signal.
**Figure S2**. **Isolation and generation of *vps13A* KO cells.** A. Schematic representation of the *vps13A* insertional mutant obtained by REMI mutagenesis, with the mutagenic plasmid pSC inserted 5′151 nt after the start codon. B. The site of insertion was identified by digestion of genomic DNA with ClaI, which allowed the recovery of the mutagenic plasmid with the genomic flanking regions of *vps13A*. We used this same plasmid to transfect WT cells in order to create a new *vps13A* KO by homologous recombination. C. Schematic representation of the *vps13A* gene in KO cells. Arrows indicate positions of the oligonucleotides used to identify KO cells. D. Identification of *vps13A* KO cells was done by PCR using distinct pairs of oligonucleotides to verify the expected size of PCR products.
**Figure S3. Phagocytosis, macropinocytosis, and intracellular killing of** ***K. pneumoniae*** **or** ***B. subtilis*** **are not defective in *vps13A* KO cells**. A. Internalization of fluorescent latex beads, of rhodamine‐labeled glutaraldehyde‐fixed K. pneumoniae and of fluorescent Dextrans in PB‐Sorbitol was assessed by flow cytometry (mean ± SEM; 3 independent experiments). Differences in phagocytosis of fixed K. pneumoniae between WT and KO cells were not significant. B. *Kp*‐GFP survival curve in WT or in *vps13A* KO cells (number of ingested bacteria is 228 for WT and 224 for *vps13A* KO cells). The set of data for WT is the same as presented in Figure 5 C.
**Figure S4**. **Vps13F is not required for growth in the presence of heat‐killed *Klebsiella*.** WT, *kil2* KO, *kil2‐vps13F* KO and *vps13F* KO cells were seeded on a lawn of heat‐killed *Klebsiella* bacteria. All cells analyzed grew comparably in these conditions.
**Figure S5. The endosomal pH in WT and in *vps13F* KO cells is similar.** To measure endosomal pH, *Dictyostelium* cells were allowed to endocytose during 18 min a mixture of dextrans coupled to Oregon Green 488 (OG, pH‐sensitive) and to Alexa 647 (A‐647, pH‐insensitive). Flow cytometry was used to measure levels of intracellular fluorescence, at different chase time points after 18 min of endocytosis. The intracellular fluorescence of both probes exhibited the same profile in WT and mutant cells. This experiment was repeated 3 times with identical results.
**Figure S6. General organization of cellular compartments is similar in *vps13F* KO and WT cells.** Immunofluorescence was used to label p25, p80, and Rhesus proteins, in order to detect distinct pericentriolar compartments, endosomes, and the contractile vacuole respectively. Confocal images are shown. Scale bar 5 μm.
**Figure S7. Western‐blot analysis of Far1 expression.** A. Western‐blot analysis of Far1 protein expression in Ax2, *far1* KO, WT (DH1) and *vps13F* KO strains. Cells were allowed to grow at a density of 3 × 10^5^ cells/ml. 1.3 × 10^6^ cells were suspended in 20 μl of 2× sample buffer and loaded on a 10% SDS‐PAGE gel. After migration and transfer of proteins on a Nitrocellulose membrane, the latter was blocked overnight with PBS‐Tween (0.1%)‐milk (7%) at 4°C. The next day, the membrane was washed twice in PBS‐Tween for 30 sec and incubated overnight at 4°C in the presence of the primary antibody (MRB168) in PBS‐Tween. The next day, after three 5‐min washes with PBS‐Tween‐milk the membrane was incubated for 2 h in the presence of the secondary antibody (HRP‐coupled anti‐mouse Ig) diluted 1/3000 in PBS‐Tween‐milk. Finally, after five washes with PBS‐Tween the ECL solution was added to reveal the presence of the Far1 protein. B. Quantification of Western‐blot analysis of SibA, Phg1A, Kil1, Kil2 and Far1 proteins in *vps13F* KO and WT strains. The relative abundance of each protein in *vps13F* KO cells and WT cells was determined in two to four independent experiments using the ImageJ software. The quantifications corresponding to gels shown in Figure 8 D are marked in red. The small increase in Kil2 levels observed in *vps13F KO* cells is not significant (*p* = 0.31; Student t‐test, *n* = 4).
**Figure S8. Isolation and generation of *kil2* KO cells.** A. Schematic representation of the *kil2* gene in WT or KO cells. To create a new *kil2* KO, we deleted 1′646 nt of the genomic sequence, 798 nt downstream of the *kil2* start codon and replaced this portion with a blasticidin resistance cassette by homologous recombination. Arrows indicate positions of the oligonucleotides used to identify KO cells. B. Identification of *kil2* KO cells was done by PCR using distinct pairs of oligonucleotides to verify both loss and gain of signals.
**Table S1**. List of species and corresponding gene accession codes used for phylogenetic analysis (Figure 2B).
