# Supplementary material for: Vps13F links bacterial recognition and intracellular killing in Dictyostelium
Source: Cell Microbiol. 2017 Feb 21;19(7):e12722. doi: 10.1111/cmi.12722 (PMC5484366; doi:10.1111/cmi.12722)

**Supplementary information**

**Figure S1.** **Isolation and generation of *vps13F* KO cells.**

A. Schematic representation of the *vps13F* insertional mutant obtained by REMI mutagenesis, with the mutagenic plasmid pSC inserted 7’144 nucleotides (nt) after the start codon.

B. The site of insertion was identified by digestion of genomic DNA with ClaI, which allowed the recovery of the mutagenic plasmid with the genomic flanking regions of *vps13F*.

C. Schematic representation of the *vps13F* gene in WT or KO cells. To create a new *vps13F* KO, we deleted 909 nt of the genomic sequence, 1’752 nt downstream of the *vps13F* start codon and replaced this portion with a blasticidin resistance cassette by homologous recombination. Arrows indicate the positions of the oligonucleotides used to identify KO cells.

D-E. Identification of *vps13F* KO cells was done by PCR using distinct pairs of oligonucleotides to verify both loss and gain of signal.

**Figure S2.** **Isolation and generation of *vps13A* KO cells.**

A. Schematic representation of the *vps13A* insertional mutant obtained by REMI mutagenesis, with the mutagenic plasmid pSC inserted 5’151 nt after the start codon.

B. The site of insertion was identified by digestion of genomic DNA with ClaI, which allowed the recovery of the mutagenic plasmid with the genomic flanking regions of *vps13A*. We used this same plasmid to transfect WT cells in order to create a new *vps13A* KO by homologous recombination.

C. Schematic representation of the *vps13A* gene in KO cells. Arrows indicate positions of the oligonucleotides used to identify KO cells.

D. Identification of *vps13A* KO cells was done by PCR using distinct pairs of oligonucleotides to verify the expected size of PCR products.

**Figure S3.** **Phagocytosis, macropinocytosis, and intracellular killing of *K. pneumoniae* or *B. subtilis* are not defective in *vps13A* KO cells.**

A. Internalization of fluorescent latex beads, of rhodamine-labeled glutaraldehyde-fixed *K. pneumoniae* and of fluorescent Dextrans in PB-Sorbitol was assessed by flow cytometry (mean ± SEM; 3 independent experiments). Differences in phagocytosis of fixed *K. pneumoniae* between WT and KO cells were not significant.

B. *Kp*-GFP survival curve in WT or in *vps13A* KO cells (number of ingested bacteria is 228 for WT and 224 for *vps13A* KO cells). The set of data for WT is the same as presented in Fig. 5C.

**Figure S4. Vps13F is not required for growth in the presence of heat-killed *Klebsiella*.**

WT, *kil2* KO, *kil2-vps13F* KO and *vps13F* KO cells were seeded on a lawn of heat-killed *Klebsiella* bacteria. All cells analyzed grew comparably in these conditions.

**Figure S5.** **The endosomal pH in WT and in *vps13F* KO cells is similar.**

To measure endosomal pH, *Dictyostelium* cells were allowed to endocytose during 18 min a mixture of dextrans coupled to Oregon Green 488 (OG, pH-sensitive) and to Alexa 647 (A-647, pH-insensitive). Flow cytometry was used to measure levels of intracellular fluorescence, at different chase time points after 18 min of endocytosis. The intracellular fluorescence of both probes exhibited the same profile in WT and mutant cells. This experiment was repeated 3 times with identical results.

**Figure S6.** **General organization of cellular compartments is similar in *vps13F* KO and WT cells.**

Immunofluorescence was used to label p25, p80, and Rhesus proteins, in order to detect distinct pericentriolar compartments, endosomes, and the contractile vacuole respectively. Confocal images are shown. Scale bar 5 μm.

**Figure S7. Western-blot analysis of Far1 expression.**

A. Western-blot analysis of Far1 protein expression in Ax2, *far1* KO, WT (DH1) and *vps13F* KO strains. Cells were allowed to grow at a density of 3x10^5^ cells/ml. 1.3x10^6^ cells were suspended in 20 μl of 2x sample buffer and loaded on a 10% SDS-PAGE gel. After migration and transfer of proteins on a Nitrocellulose membrane, the latter was blocked overnight with PBS-Tween (0.1%)-milk (7%) at 4°C. The next day, the membrane was washed twice in PBS-Tween for 30 sec and incubated overnight at 4°C in the presence of the primary antibody (MRB168) in PBS-Tween. The next day, after three 5-min washes with PBS-Tween-milk the membrane was incubated for 2 h in the presence of the secondary antibody (HRP-coupled anti-mouse Ig) diluted 1/3000 in PBS-Tween-milk. Finally, after five washes with PBS-Tween the ECL solution was added to reveal the presence of the Far1 protein.

B. Quantification of Western-blot analysis of SibA, Phg1A, Kil1, Kil2 and Far1 proteins in *vps13F* KO and WT strains. The relative abundance of each protein in *vps13F* KO cells and WT cells was determined in two to four independent experiments using the ImageJ software. The quantifications corresponding to gels shown in Fig. 8D are marked in red. The small increase in Kil2 levels observed in *vps13F KO* cells is not significant (p=0.31; Student t-test, n=4).

**Figure S8. Isolation and generation of *kil2* KO cells.**

A. Schematic representation of the *kil2* gene in WT or KO cells. To create a new *kil2* KO, we deleted 1’646 nt of the genomic sequence, 798 nt downstream of the *kil2* start codon and replaced this portion with a blasticidin resistance cassette by homologous recombination. Arrows indicate positions of the oligonucleotides used to identify KO cells.

B. Identification of *kil2* KO cells was done by PCR using distinct pairs of oligonucleotides to verify both loss and gain of signals.

**Table S1. List of species and corresponding gene accession codes used for phylogenetic analysis (Fig. 2B).**

| **Protein** | **Organism** | **Accession ID** |
| --- | --- | --- |
| Vps13A | *D. discoideum* | XP_637643 |
| Vps13B | *D. discoideum* | XP_644147 |
| Vps13C | *D. discoideum* | XP_647570 |
| Vps13D | *D. discoideum* | XP_637675 |
| Vps13E | *D. discoideum* | XP_647037 |
| Vps13F | *D. discoideum* | XP_637397 |
| Vps13A | *D. purpureum* | XP_003286357 |
| Vps13B | *D. purpureum* | XP_003292820 |
| Vps13C | *D. purpureum* | XP_003293812 |
| Vps13D | *D. purpureum* | XP_003290022 |
| Vps13E | *D. purpureum* | XP_003291063 |
| Vps13F | *D. purpureum* | XP_003293882 |
| Vps13A | *D. fasciculatum* | XP_004358813 |
| Vps13B | *D. fasciculatum* | XP_004363054 |
| Vps13C | *D. fasciculatum* | XP_004361633 |
| Vps13D | *D. fasciculatum* | XP_004358786 |
| Vps13E | *D. fasciculatum* | XP_004359975 |
| Vps13F | *D. fasciculatum* | XP_004358101 |
| Vps13A | *Homo sapiens* | NP_150648 |
| Vps13B | *Homo sapiens* | NP_060360 |
| Vps13C | *Homo sapiens* | NP_065872 |
| Vps13D | *Homo sapiens* | NP_056193 |
| Vps13A | *Mus musculus* | NP_766616 |
| Vps13B | *Mus musculus* | NP_796125 |
| Vps13C | *Mus musculus* | NP_796158 |
| Vps13D | *Mus musculus* | NP_001263431 |
| Vps13A | *Danio rerio* | NP_001112365 |
| Vps13C | *Danio rerio* | XP_009301518 |
| Vps13D | *Danio rerio* | XP_001919988 |
| Vps13 | *Caenorhabditis elegans* | NP_740899 |
| Vps13A | *Drosophila melanogaster* | NP_610299 |
| Vps13B | *Drosophila melanogaster* | NP_729825 |
| Vps13C | *Drosophila melanogaster* | NP_651753 |
| Vps13 | *Saccharomyces cerevisiae* | NP_013060 |
| Vps13 | *Cryptococcus neoformans* | XP_012052436 |
| Vps13 | *Neurospora crassa* | XP_960097 |

**Figure S1:**


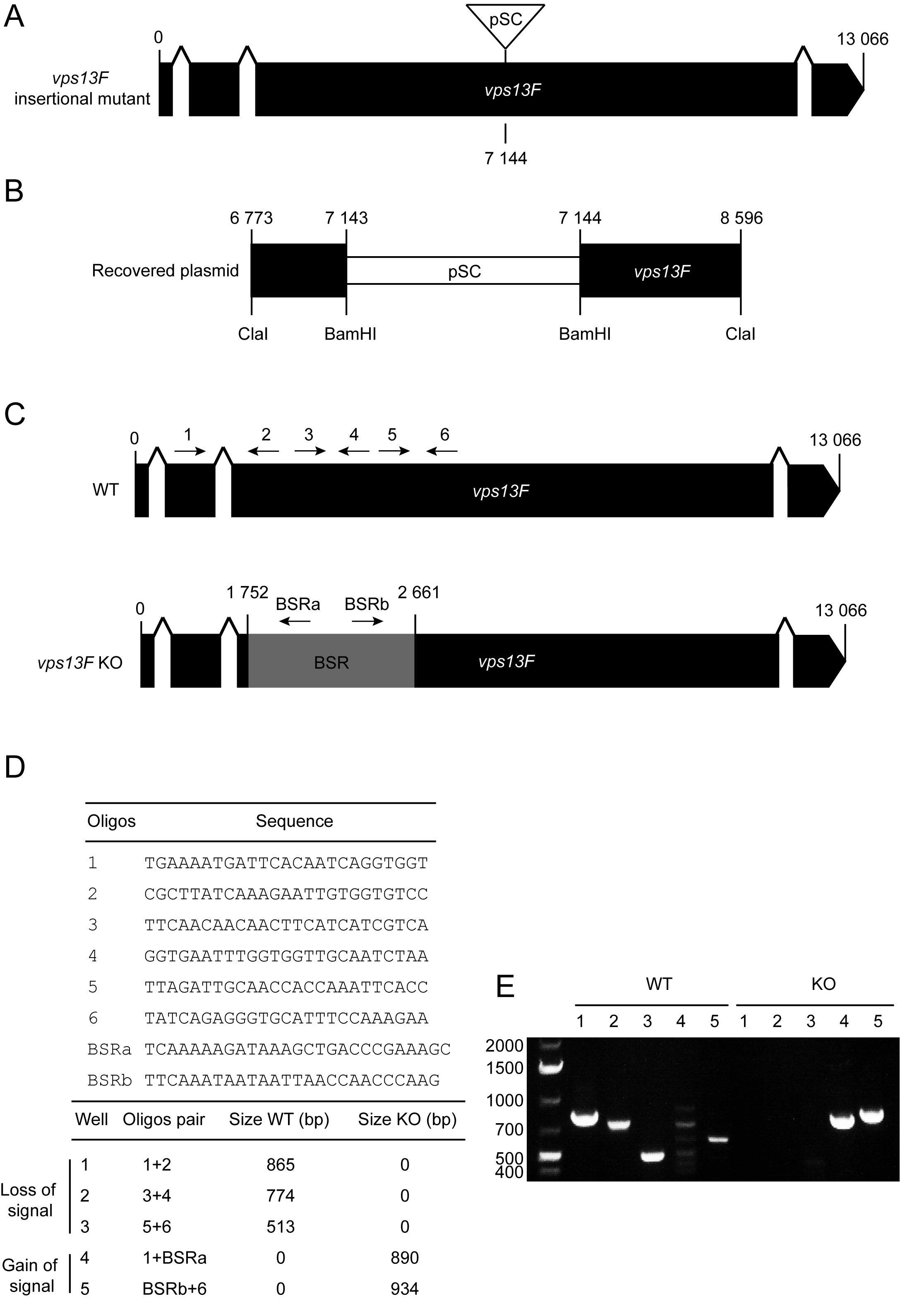


**Figure S2:**


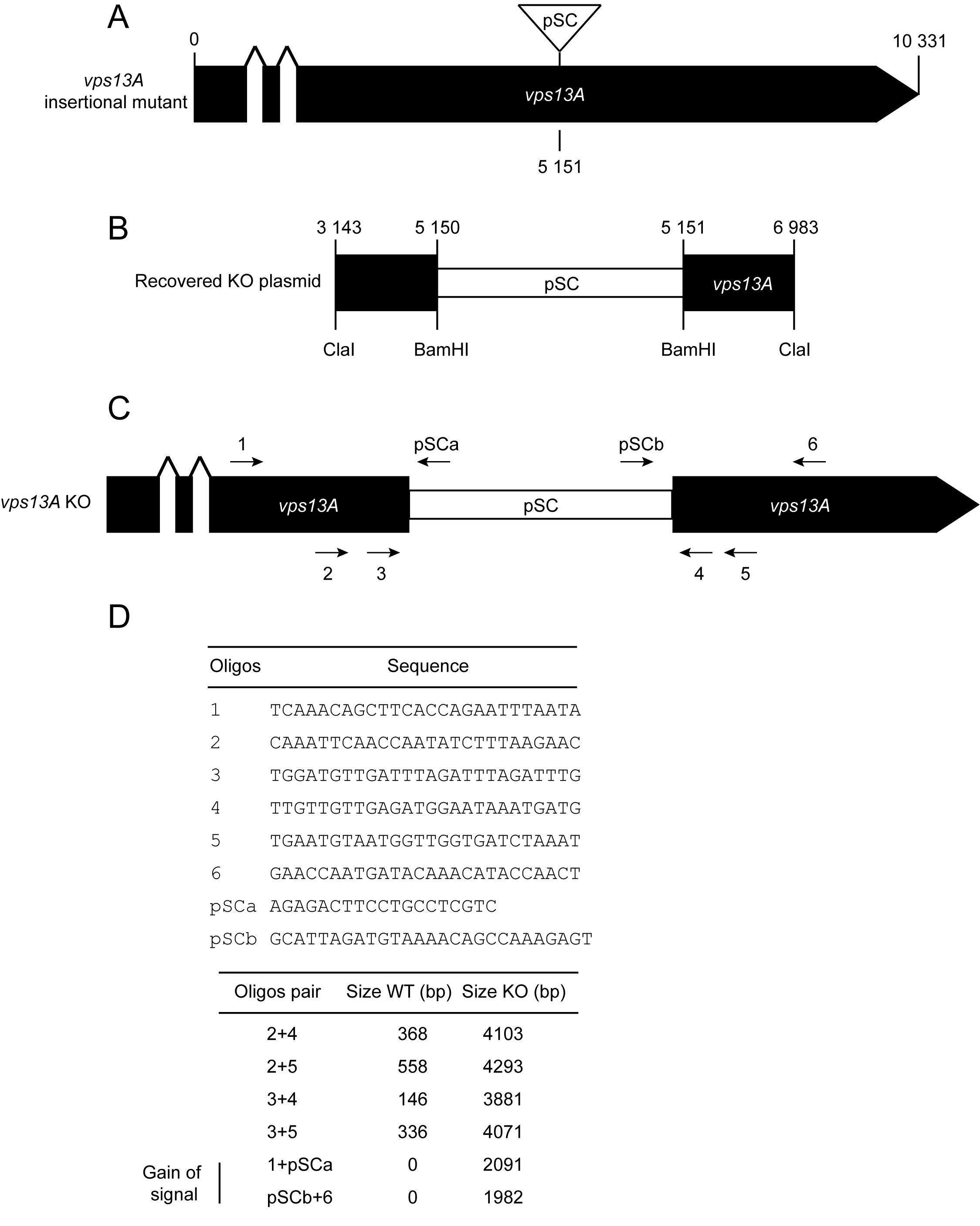


**Figure S3:**


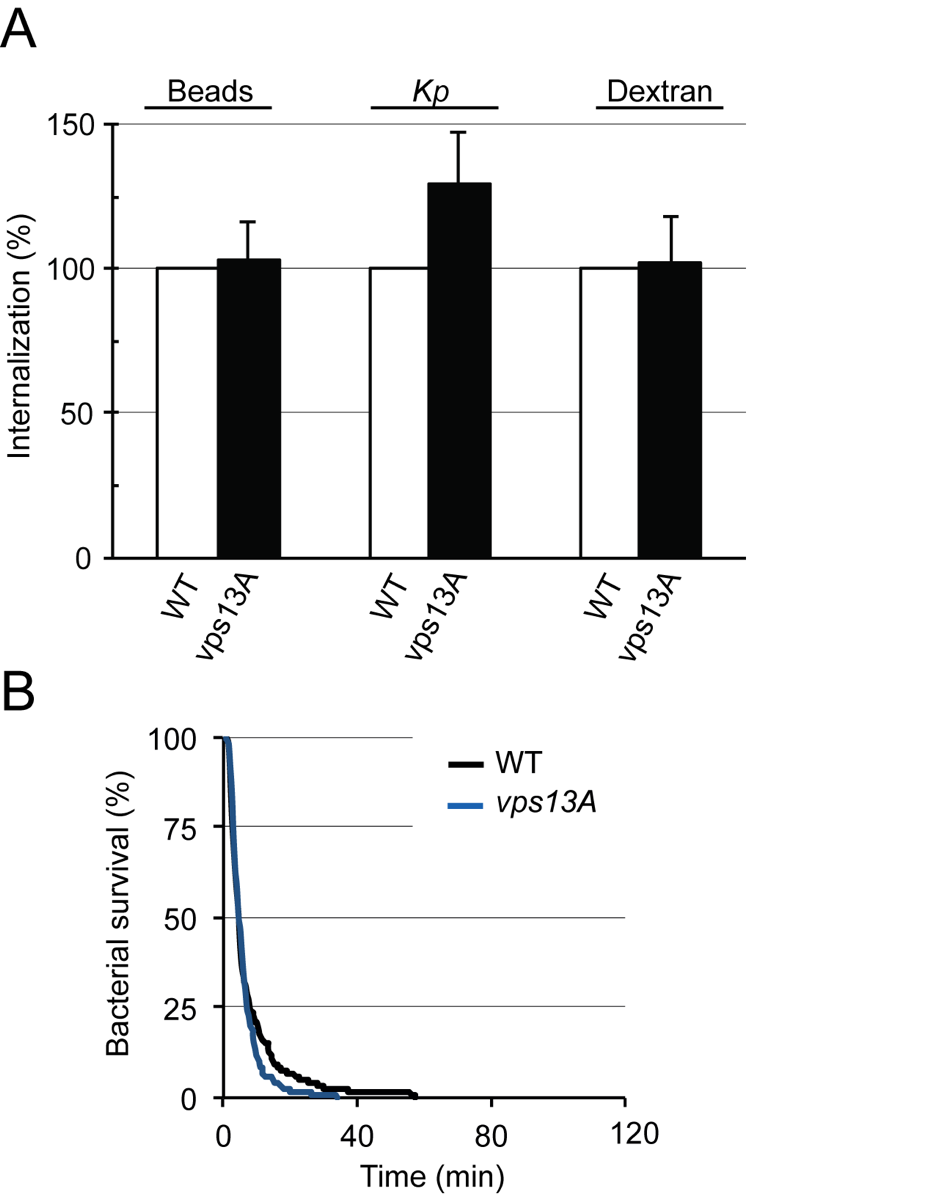


**Figure S4:**


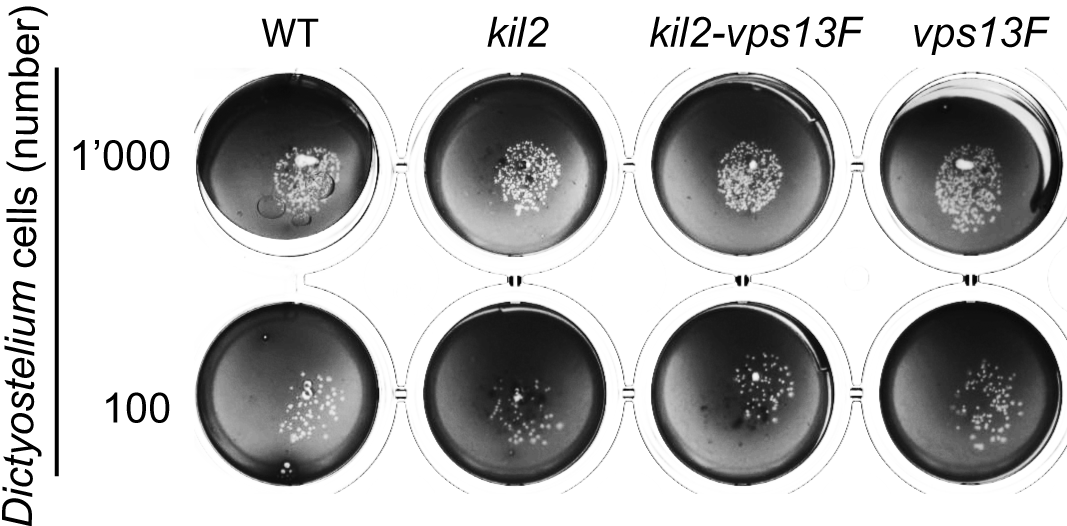


**Figure S5:**


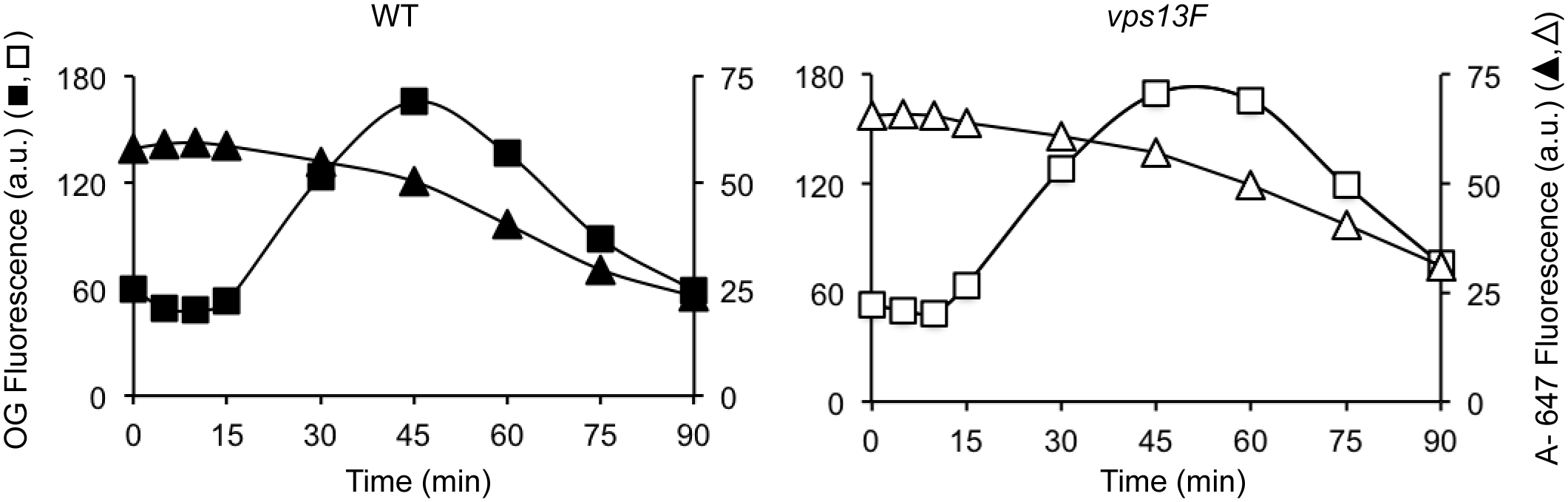


**Figure S6:**


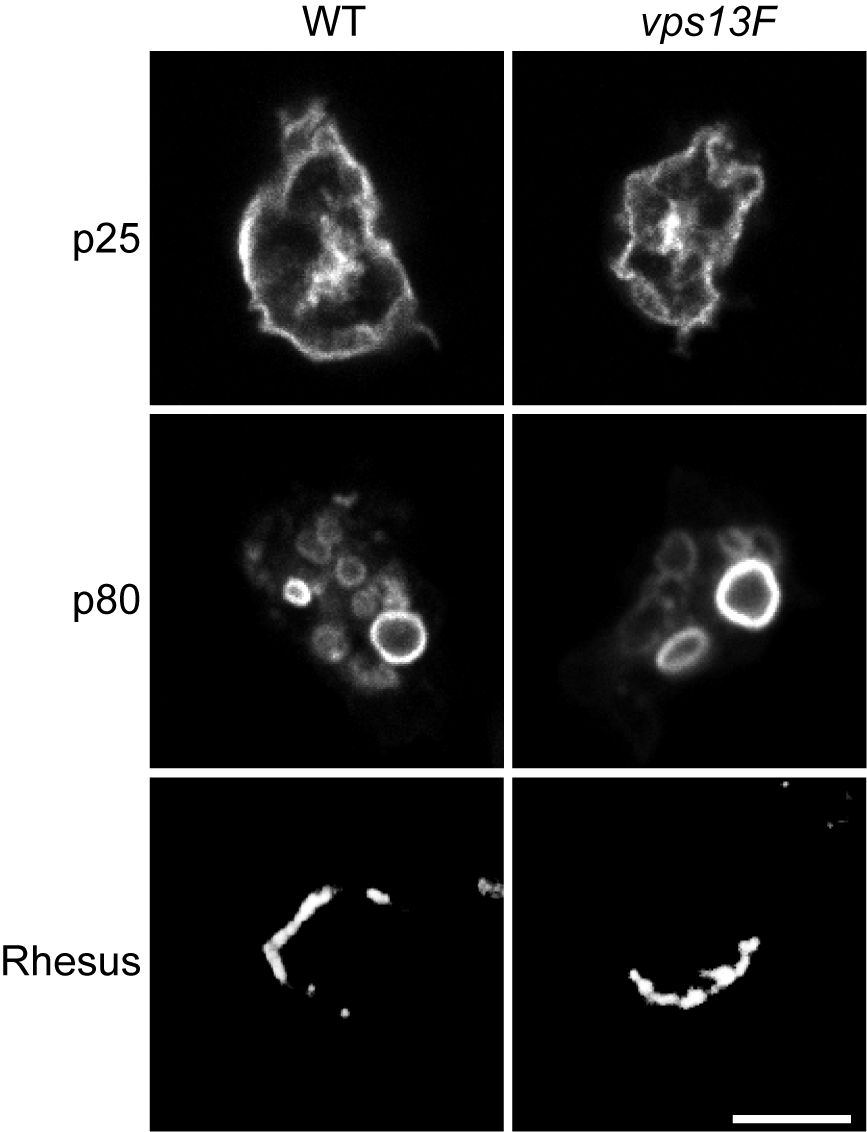


**Figure S7:**


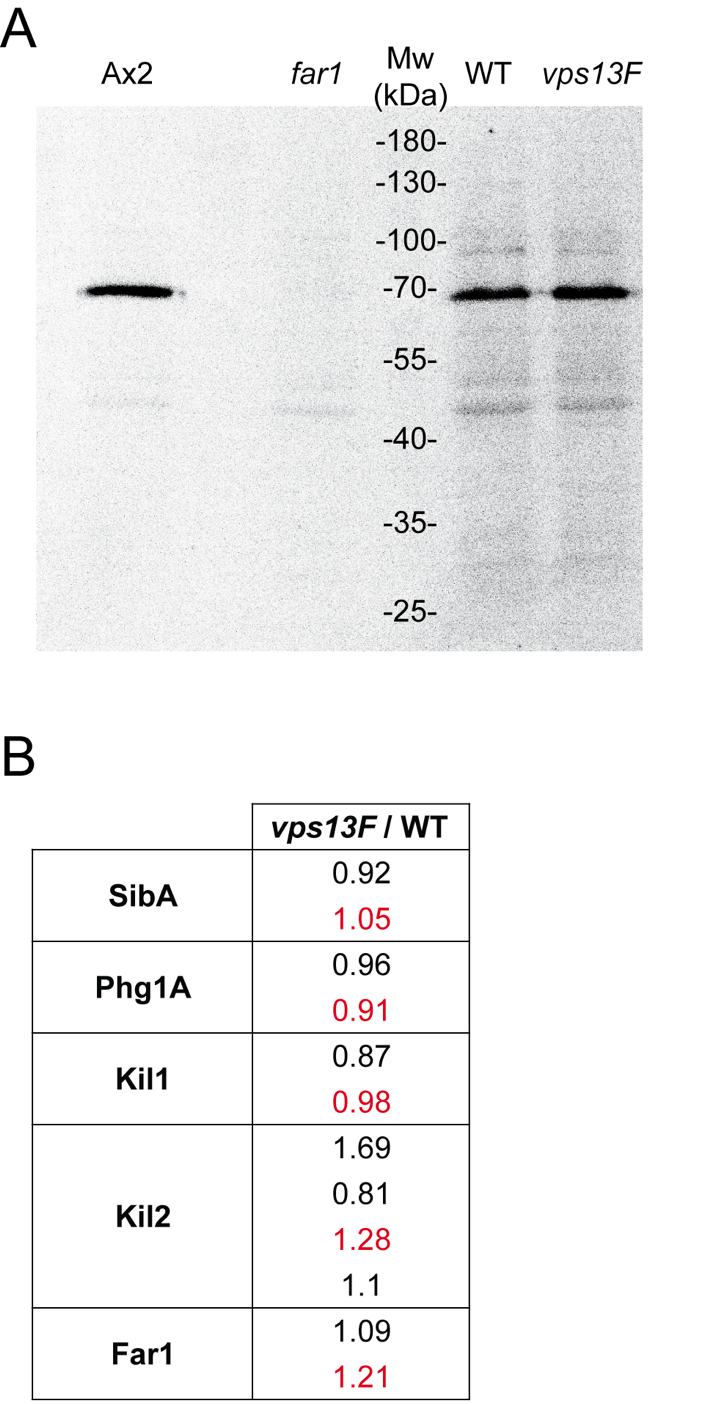


**Figure S8:**


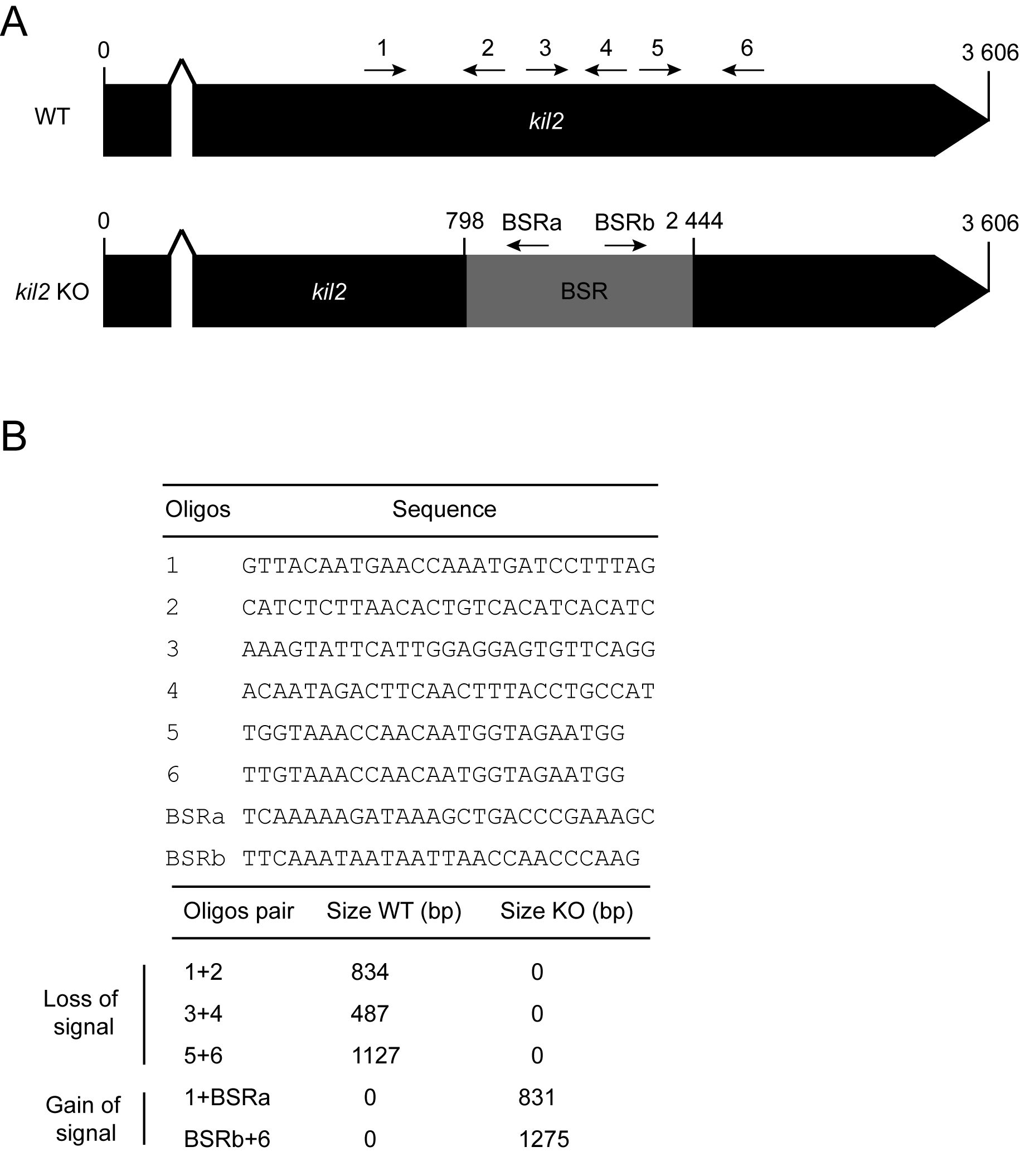

Supplement: Supplementary file 1 — Figure S1. Isolation and generation of vps13F KO cells. A. Schematic representation of the vps13F insertional mutant obtained by REMI mutagenesis, with the mutagenic plasmid pSC inserted 7′144 nucleotides (nt) after the start codon. B. The site of insertion was identified by digestion of genomic DNA with ClaI, which allowed the recovery of the mutagenic plasmid with the genomic flanking regions of vps13F. C. Schematic representation of the vps13F gene in WT or KO cells. To create a new vps13F KO, we deleted 909 nt of the genomic sequence, 1′752 nt downstream of the vps13F start codon and replaced this portion with a blasticidin resistance cassette by homologous recombination. Arrows indicate the positions of the oligonucleotides used to identify KO cells. D‐E. Identification of vps13F KO cells was done by PCR using distinct pairs of oligonucleotides to verify both loss and gain of signal. Figure S2. Isolation and generation of vps13A KO cells. A. Schematic representation of the vps13A insertional mutant obtained by REMI mutagenesis, with the mutagenic plasmid pSC inserted 5′151 nt after the start codon. B. The site of insertion was identified by digestion of genomic DNA with ClaI, which allowed the recovery of the mutagenic plasmid with the genomic flanking regions of vps13A. We used this same plasmid to transfect WT cells in order to create a new vps13A KO by homologous recombination. C. Schematic representation of the vps13A gene in KO cells. Arrows indicate positions of the oligonucleotides used to identify KO cells. D. Identification of vps13A KO cells was done by PCR using distinct pairs of oligonucleotides to verify the expected size of PCR products. Figure S3. Phagocytosis, macropinocytosis, and intracellular killing of K. pneumoniae or B. subtilis are not defective in vps13A KO cells. A. Internalization of fluorescent latex beads, of rhodamine‐labeled glutaraldehyde‐fixed K. pneumoniae and of fluorescent Dextrans in PB‐Sorbitol was assessed by flow cytometry [file CMI-19-na-s001.doc]
